# Risk willingness in multiple system atrophy and Parkinson’s disease understanding patient preferences

**DOI:** 10.1038/s41531-024-00764-5

**Published:** 2024-08-15

**Authors:** Alexander Maximilian Bernhardt, Marc Oeller, Isabel Friedrich, Emre Kocakavuk, Eliana Nachman, Kevin Peikert, Malte Roderigo, Andreas Rossmann, Tabea Schröter, Lea Olivia Wilhelm, Tino Prell, Christoph van Riesen, Johanna Nieweler, Sabrina Katzdobler, Markus Weiler, Heike Jacobi, Tobias Warnecke, Inga Claus, Carla Palleis, Stephan Breimann, Björn Falkenburger, Moritz Brandt, Andreas Hermann, Jost-Julian Rumpf, Joseph Claßen, Günter Höglinger, Florin Gandor, Johannes Levin, Armin Giese, Annette Janzen, Wolfgang Hermann Oertel

**Affiliations:** 1 Department of Neurology, Ludwig-Maximilians-Universität München, Munich, Germany; 2 German Center for Neurodegenerative Diseases (DZNE), Munich, Germany; 3 Department for Proteomics and Signal Transduction, Max Planck Institute of Biochemistry, Martinsried, Germany; 4 Department of Neurology, University of Leipzig Medical Center, Leipzig, Germany; 5 Department of Hematology and Stem Cell Transplantation, West German Cancer Center, University Hospital Essen, Essen, Germany; 6 VIB Center for Brain & Disease Research, Leuven, Belgium; 7 KU Leuven Department of Neurosciences, Leuven Brain Institute, Mission Lucidity, Leuven, Belgium; 8 Translational Neurodegeneration Section “Albrecht Kossel”, Department of Neurology, University Medical Center Rostock, University of Rostock, Rostock, Germany; 9 Center for Transdisciplinary Neurosciences Rostock (CTNR), University Medical Center Rostock, Rostock, Germany; 10 United Neuroscience Campus Lund-Rostock (UNC), Rostock, Germany; 11 Department of Neurology with Institute of Translational Neurology, University of Muenster, Muenster, Germany; 12 Department of Cardiology, Augustinum Klinik München, München, Germany; 13 Department of Neurology, Jena University Hospital, Jena, Germany; 14 Medical School Berlin, Berlin, Germany; 15 Department of Geriatrics, Halle University Hospital, Halle, Germany; 16 Department of Neurology, University Medical Center Göttingen, Göttingen, Germany; 17 German Center for Neurodegenerative Diseases (DZNE), Göttingen, Germany; 18 Department of Neurology, Heidelberg University Hospital, Heidelberg, Germany; 19 Department of Neurology and Neurorehabilitation, Hospital Osnabrück, Osnabrück, Germany; 20 Munich Cluster for Systems Neurology (SyNergy), Munich, Germany; 21 Department of Bioinformatics, Wissenschaftszentrum Weihenstephan, Technical University of Munich, Freising, Germany; 22 Metabolic Biochemistry, Ludwig-Maximilians-University Munich, Munich, Germany; 23 Department of Neurology, TU Dresden, Dresden, Germany; 24 Deutsches Zentrum für Neurodegenerative Erkrankungen (DZNE), Dresden, Germany; 25 German Center for Neurodegenerative Diseases (DZNE), Rostock/Greifswald, Germany; 26 Movement Disorders Clinic, Beelitz-Heilstätten, Germany; 27 Department of Neurology, Otto-von-Guericke University, Magdeburg, Germany; 28 MODAG GmbH, Wendelsheim, Germany; 29 Center for Neuropathology and Prion Research, Ludwig-Maximilians-University Munich, Munich, Germany; 30 Department of Neurology, Philipps-University Marburg, Marburg, Germany; 31 Institute for Neurogenomics, Helmholtz Center for Environment and Health, München, Germany

**Keywords:** Parkinson's disease, Pharmaceutics, Drug development

## Abstract

Disease-modifying therapeutics in the α-synucleinopathies multiple system atrophy (MSA) and Parkinson’s Disease (PD) are in early phases of clinical testing. Involving patients’ preferences including therapy-associated risk willingness in initial stages of therapy development has been increasingly pursued in regulatory approval processes. In our study with 49 MSA and 38 PD patients, therapy-associated risk willingness was quantified using validated standard gamble scenarios for varying severities of potential drug or surgical side effects. Demonstrating a non-gaussian distribution, risk willingness varied markedly within, and between groups. MSA patients accepted a median 1% risk [interquartile range: 0.001–25%] of sudden death for a 99% [interquartile range: 99.999–75%] chance of cure, while PD patients reported a median 0.055% risk [interquartile range: 0.001–5%]. Contrary to our hypothesis, a considerable proportion of MSA patients, despite their substantially impaired quality of life, were not willing to accept increased therapy-associated risks. Satisfaction with life situation, emotional, and nonmotor disease burden were associated with MSA patients’ risk willingness in contrast to PD patients, for whom age, and disease duration were associated factors. An individual approach towards MSA and PD patients is crucial as direct inference from disease (stage) to therapy-associated risk willingness is not feasible. Such studies may be considered by regulatory agencies in their approval processes assisting with the weighting of safety aspects in a patient-centric manner. A systematic quantitative assessment of patients’ risk willingness and associated features may assist physicians in conducting individual consultations with patients who have MSA or PD by facilitating communication of risks and benefits of a treatment option.

## Introduction

Multiple System Atrophy (MSA) is an invariably fatal α-synucleinopathy characterized by fast disease progression with no effective therapy available^1^. In contrast, patients with Parkinson’s disease (PD), which is a related, but less rapidly progressive α-synucleinopathy, have access to symptomatic therapies such as effective pharmacotherapy or deep brain stimulation to alleviate symptoms. Novel disease-modifying mainly pharmacotherapeutic strategies in both α-synucleinopathies are on the verge of or in early phase of being clinically tested^2,3^.

Involving patients’ preferences from the initial stages of therapy development has been increasingly pursued by regulatory agencies such as the US Food and Drug Administration^4^ and the European Medicines Agency^5^. Patient centricity plays an essential role in shared decision-making of referral and treatment decisions in clinical routine. These decisions consider the physician’s prognosis assessment, evaluation of potential benefits and risks associated with different treatment options, and the patient’s values and needs. However, patients may have different perceptions of the benefits and risks of planned therapy due to interindividual variations in risk/benefit perceptions, and these differences may exist independently of the severity or type of disease.

For evaluating patient preferences in situations involving risk, the Standard Gamble (SG) is the preferred method^6^. This instrument directly obtains preferences from patients and is the most theoretically valid way of eliciting preferences^7,8^. This study aimed to quantitatively assess the potential risks related to disease-modifying therapeutic options that patients with MSA and PD would be willing to accept in a clinical trial. The SG method was used to cover a broad range of risks, including permanent bearable, severe, and lethal drug side effects or surgery complications. The study also aimed to identify important associated factors for accepting therapy-associated risks. It was hypothesized that MSA patients, who have limited symptomatic treatment options, and shorter survival, would demonstrate a higher willingness to accept risks compared to PD patients, who benefit from established symptomatic treatments, resulting in longer survival, and better quality of life.

## Results

### Participants

Detailed demographic and clinical data are provided in Table 1 and in Supplementary Table 1. At time of assessment, disease duration was shorter in the MSA than in the PD group (2.1 ± 1.7 years vs 8.3 ± 5.5 years). With regard to motor functioning, MSA patients were more impaired than PD patients as reflected by MDS-UPDRS II (24.0 ± 8.9 vs 11.8 ± 4.6), MDS-UPDRS III (43.6 ± 12.2 vs 23.3 ± 9.0) and Hoehn and Yahr stage values (3.6 ± 0.9 vs 2.7 ± 0.8). These differences were reflected in lower Quality of life (QoL), motor, nonmotor, and emotional subscores, and lower satisfaction with life situations in MSA patients. Beneficial response to Levodopa treatment (as assessed anamnestically) occurred in all PD patients but in only 25.5% of MSA patients. The two groups did not significantly differ regarding education levels, depression, age, and sex (Table 1). We specifically assessed the personality feature of being a risk-taker or risk-avoider using the validated Risk Propensity Scale (RPS) and found no differences between MSA and PD patients (*p* = 0.406, Wilcoxon). Mean RPS number score was 17.8 ± 6.2, indicating an overall aversion to taking risks. Reasons for and against participating in clinical trials were similar between MSA and PD patients (Supplementary Fig. 1): The statements with the highest agreement when considering participation in a clinical trial were: “The trial’s results help other patients suffering from the same illness” (MSA 90%, PD 95%), “The trial advances research” (MSA 86%, PD 85%), and “Having sufficient information about the trial” (MSA 78%, PD 85%). The most crucial barriers for participation in a clinical trial were identified as “Unpleasant interventions” (MSA 51%, PD 61%), and “Physical fitness makes journey burdensome” (MSA 65%, PD 59%).

**Table 1:** Clinical and demographic characteristics of the study cohort

	Overall		MSA overall		PD		*p* (MSA vs PD)
*N* (%)	87	(100.0)	49	(100.0)	38	(100.0)	
MSA-P			28	(57.1)			
MSA-C			21	(42.9)			
Diagnostic certainty							
Possible, *n* (%)			10	(20.4)			
Probable, *n* (%)			39	(79.6)			
Sex							0.11
Women, *n* (%)	38	(43.7)	25	(51.0)	14	(36.8)	
Men, *n* (%)	49	(56.3)	24	(49.0)	24	(63.2)	
Age							
Age at study entry, mean (SD)	65.0	(9.8)	63.7	(8.8)	66.5	(10.7)	0.19
Age at primary diagnosis, mean (SD)	60.4	(10.7)	61.8	(8.9)	58.5	(12.4)	0.16
Time since primary diagnosis in years, mean (SD)	4.8	(4.9)	2.1	(1.7)	8.3	(5.5)	>0.001
Hoehn and Yahr stage, mean (SD)	3.2	(1.0)	3.6	(0.9)	2.7	(0.8)	>0.001
Stage 1, *n* (%)	2	(2.3)	0	(0.0)	2	(5.3)	
Stage 2, *n* (%)	19	(21.8)	4	(8.2)	15	(39.5)	
Stage 3, *n* (%)	40	(46.0)	23	(46.9)	17	(44.7)	
Stage 4, *n* (%)	15	(17.2)	12	(24.5)	3	(7.8)	
Stage 5, *n* (%)	11	(12.6)	10	(20.4)	1	(2.6)	
Scales							
UPDRS I mean (SD)	2.7	(2.0)	2.7	(2.6)	2.7	(2.1)	0.82
UPDRS II mean (SD)	14.3	(7.5)	24.0	(8.9)	11.8	(4.6)	>0.001
UPDRS III mean (SD)	30.4	(14.0)	43.6	(12.2)	23.3	(9.0)	>0.001
UMSARS I mean (SD)			20.3	(6.8)			
UMSARS II mean (SD)			21.6	(7.6)			
Treatment							
Levodopa treatment, *n* (%)	65	(74.7)	27	(55.1)	38	(100.0)	0.85
Dopamine agonist treatment, *n* (%)	21	(24.1)	1	(2.0)	20	(52.6)	>0.001
Levodopa response							>0.001
Beneficial response, *n* (%)	51	(58.6)	13	(26.5)	38	(100.0)	
No or poor response, *n* (%)	28	(32.2)	28	(57.1)	0	(2.3)	
Unknown, *n* (%)	8	(9.2)	8	(16.3)	0	(0.0)	
Deep brain stimulation, *n* (%)	9	(10.3)	2	(4.1)	7	(18.4)	0.049
Secondary education							0.67
≤12 years, *n* (%)	37	(42.5)	23	(46.9)	14	(36.8)	
>12 years, *n* (%)	49	(56.3)	26	(53.1)	23	(60.5)	
Unknown, *n* (%)	1	(1.1)	0	(0.0)	1	(2.6)	
Quality of life							
Motor subscore, mean (SD)	58.0	(23.5)	45.1	(20.9)	74.7	(14.4)	>0.001
Nonmotor subscore, mean (SD)	63.4	(15.6)	58.5	(15.2)	69.6	(14.1)	>0.001
Emotional subscore, mean (SD)	67.9	(20.4)	58.9	(18.9)	79.7	(15.7)	>0.001
Satisfaction with life situation, mean (SD)	47.1	(24.7)	37.6	(24.2)	60.1	(18.9)	>0.001
Risk propensity score, mean (SD)	17.8	(6.2)	17.3	(6.7)	18.4	(5.4)	0.41
Depression present, *n* (%)	30	(34.5)	17	(34.7)	13	(34.2)	0.92

### Individuals’ views on specific therapy-associated risks

**Fig. 1:**
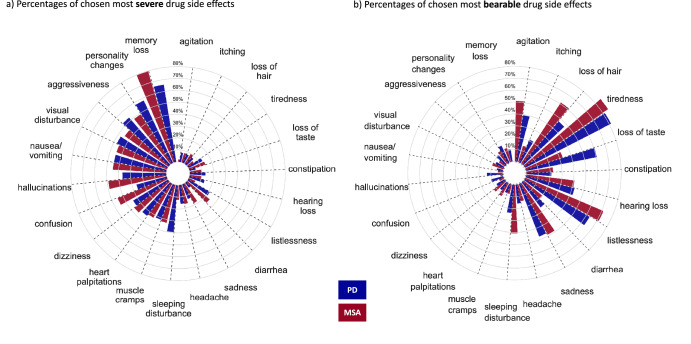
Patients’ attitudes towards drug-related risks. Patients were presented with a priori-defined lists of possible side effects of drugs that they would suffer from permanently. They were asked to tick the three cognitive and emotional as well as the three physical side effects that they considered to be most severe (**a**) or most bearable (**b**). Percentages of chosen side effects are displayed.

We next sought to evaluate MSA and PD patients’ perception on different common a priori defined side effects of hypothetical medication or surgery procedures. Percentages of chosen side effects are displayed in Fig. 1 for drug side effects and Supplementary Fig. 2 for surgery complications. Cognitive and emotional drug side effects were rated by both groups as most severe: MSA patients most frequently chose memory loss (80%), personality changes and hallucinations (both 50%), PD patients chose memory loss (66%), personality changes (58%) and aggressiveness (53%). Visual disturbance (MSA 45%, PD 47%) and nausea/vomiting (both 45%) were chosen as the most severe physical side effects. PD patients feared sleeping disturbances more (40% vs 12%, *p* = 0.007, χ²) and diarrhea less (5% vs 25%, *p* = 0.03, χ²). Tiredness (MSA 86%, PD 82%) and listlessness (MSA 71%, PD 63%) were named most frequently as the most bearable side effects in both groups, whereas PD patients accepted loss of taste more often as a most bearable side effect (MSA: 33%, PD 61%, *p* = 0.02, χ²).

### Quantification of individuals’ willingness to accept specific therapy-associated risks

**Fig. 2:**
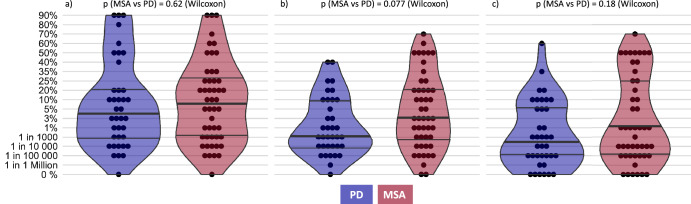
Patients’ willingness to take risks regarding severity of drug side effects. Individuals’ answers to standard gamble (SG) scenarios are shown. Each dot represents one patient and his or her maximum accepted risk for the respective scenario. Horizontal lines show median and interquartile ranges. No significant group differences between MSA and PD regarding the 3 SG could be detected (Wilcoxon-tests). **a** Maximum accepted risk of most bearable drug side effects. Side effects such as tiredness and listlessness were most commonly reported as bearable. Tiredness was noted by 86% of MSA patients and 82% of PD patients, while listlessness was reported by 71% of MSA and 63% of PD patients. Other minor side effects like loss of taste were considered bearable more frequently by PD patients (61%) compared to MSA patients (33%). **b** Maximum accepted risk of most severe drug side effects. This category includes both cognitive and emotional as well as physical drug side effects that significantly affect quality of life. Cognitive and emotional side effects deemed most severe included memory loss (MSA: 80%, PD: 66%), personality changes (MSA: 50%, PD: 58%), and hallucinations or aggressiveness. Physical side effects such as visual disturbances and nausea/vomiting were commonly rated severe by both groups (MSA: 45%, PD: 47%). PD patients particularly noted sleep disturbances as severe (40%) compared to MSA patients (12%). **c** Maximum accepted risk of fatal adverse reaction. Represents the ultimate risk, which is death.

Based on the selected specific side effects, Fig. 2 illustrates individuals’ maximum accepted percent probabilities of permanent severe or bearable drug side effects and death. Notably, individuals’ answers were not normally distributed and varied widely for most scenarios. MSA patients displayed a (non-significant) trend to higher therapy-associated risk willingness than PD patients (lethal side effects: *p* = 0.18, severe side effects: *p* = 0.077, bearable side effects: *p* = 0.62, Wilcoxon): they would accept a median 1% risk [interquartile range: 0.001–25%] of sudden death to cure their symptoms for a 99% [interquartile range: 99.999–75%] chance of cure whereas PD patients reported a median 0.055% risk [interquartile range: 0.001–5%]. Higher levels of risks were tolerated regarding most severe drug side effects (MSA median: 3% [interquartile range: 0.08–20%] risk for a 97% [interquartile range: 99.92–80%] chance of cure, PD median: 0.1% [interquartile range: 0.01–10%] risk) and even higher levels regarding most bearable drug side effects (MSA median: 7,5% [interquartile range: 0.1–27.5%] risk for a 92.5% [interquartile range: 99.9–72.5%] chance of cure, PD median 3% [interquartile range: 0.08–20%] risk). One patient each with MSA and PD declined to take any risks, even if the side effects of the drug were the most bearable. Data on surgery complications are shown in Supplementary Fig. 3.

### Features associated with risk decision-making for investigational drugs are different between MSA and PD patients

**Fig. 3:**
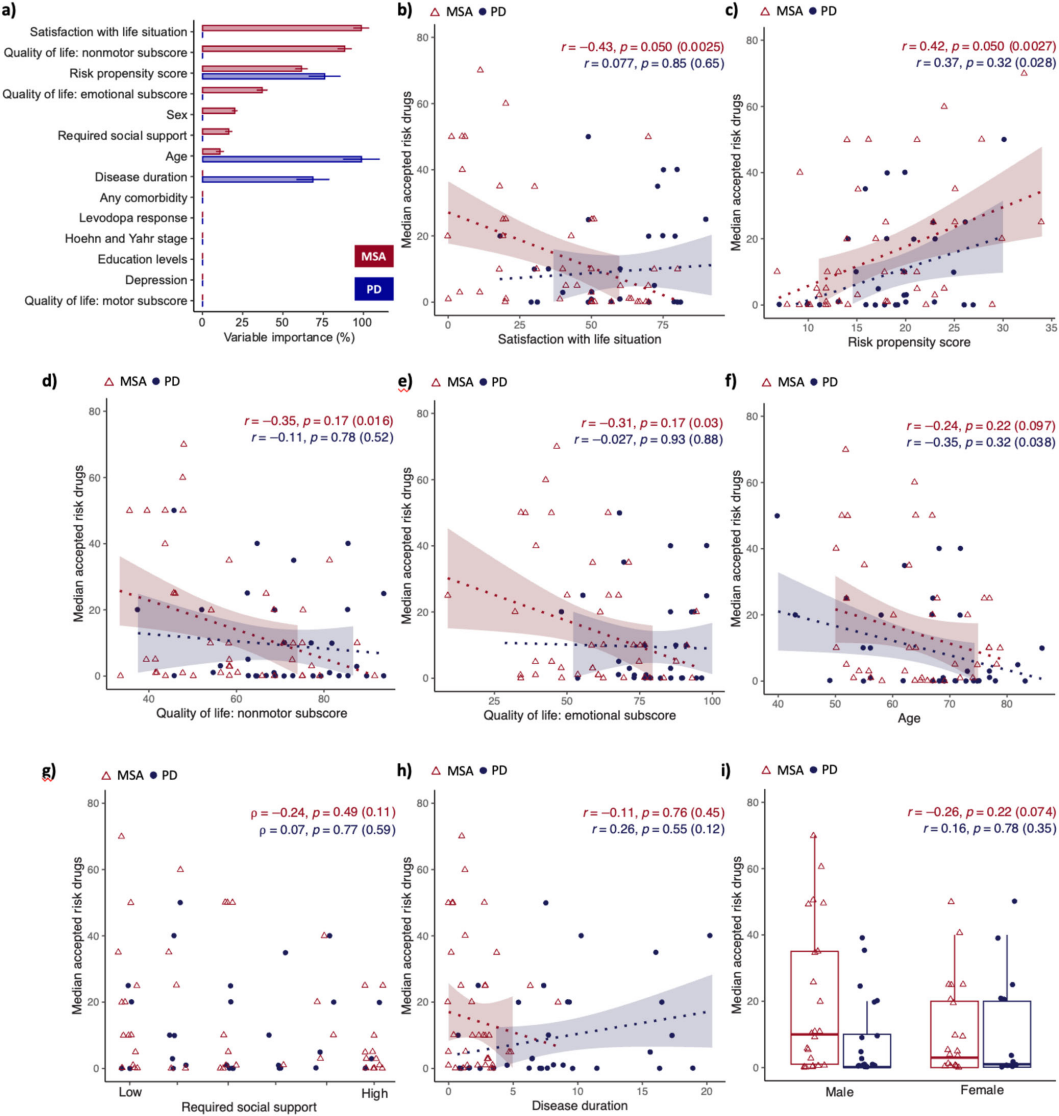
Variable importance of clinical and psychosocial features in association with risk decision making for investigational drugs. In (**a**) the x-axis displays the conditional variable importance obtained from random forest regression analyses conducted separately for MSA (red) and PD (blue) patients. Shown are point estimates and 95% confidence intervals obtained from 200 runs. The variable with the highest importance is assigned a value of 100%, and all other variables are expressed as a percentage relative to that value. Any variables with confidence intervals that include zero or negative values are considered to have no predictive power in our model and are assigned a value of zero. In (**b**–**i**) bivariate associations between clinical, psychosocial features, and the median accepted risk of drug side effects are presented. MSA and PD patients are represented as red triangles and blue dots, respectively. Pearson correlation coefficients (“*r*”) were used to analyze continuous variables such as satisfaction with life situation (**b**), RPS (**c**), quality of life: nonmotor subscore (**d**), quality of life: emotional subscore (**e**), age (**f**), required social support (**g**), disease duration (**h**) and sex (**i**). Point-biserial correlation (“*r*”) was used for categorical variables, and Spearman correlation coefficients (“*ρ*”) were employed for ordinal scaled variables like the degree of required social support. Regression curves and 95% confidence intervals are provided for continuous variables. *P* values (“*p*”), both raw and adjusted following the Benjamini-Hochberg procedure, were computed (raw *p* values are shown in brackets).

To estimate the contribution of each feature, we employed random forest regression to compute conditional variable importance values (Fig. 3a). Regarding the median accepted risk of drug side effects, MSA patients revealed a different set of associated features when compared to PD patients: in descending order of importance, overall satisfaction with life situation, nonmotor QoL subscore, RPS, emotional QoL subscore, degree of required social support, and age. Conversely, the median acceptable risk of drug side effects in PD patients was associated with age, RPS, and disease duration. Unlike in MSA patients, nonmotor, and emotional QoL subscores, degree of required social support, and satisfaction with life situation did not emerge as independent associated features in PD patients. Additionally, in both groups, variables such as the presence of any comorbidity, response to levodopa, depression, education levels, motor QoL subscore, and Hoehn and Yahr stage did not manifest as independent associated features. The impact of sex on risk willingness is nuanced and scenario-dependent, varying significantly between drug and surgery side effects and across patient groups. The model explained 51.97% of the variance in median accepted risk of drug side effects for MSA patients (R squared) and 25.29% for PD patients, as determined by 10-fold cross-validation.

To further understand the effects of associated features, bivariate associations between associated features, and median accepted drug risk were calculated (Fig. 3b–i, Supplementary Fig. 9). MSA patients with highest median accepted risk of drug side effects suffered more likely from a low satisfaction with life situation, low nonmotor, and emotional QoL subscores, did not require a high degree of social support, and were relatively young. They likely exhibited a relatively pronounced personality feature of being risk-takers (i.e., high RPS). PD patients with higher median acceptable risk of drug side effects were likely to be relatively young, have a pronounced overall risk-taking attitude, and have had a relatively long disease duration. Despite no significant association between attitudes or intentions towards clinical trials and risk willingness, a clear trend indicated that participants with higher intentions to participate were more inclined to accept greater therapy-associated risks (Supplementary Figs. 9 and 10). Features associated with risk willingness regarding surgery complications are shown in Supplementary Figs. 6 and 10. Individual-level analyses can be found in Supplementary Figs. 4, 5, 7, and 8. While the results did not reach statistical significance, our analysis revealed a clear trend where MSA-C patients exhibited a lower willingness to accept specific therapy-associated risks compared to MSA-P patients (Supplementary Fig. 11). This trend is consistent with theories suggesting that cerebellar dysfunction, particularly prevalent in MSA-C, may impact emotional regulation and decision-making processes^9,10^.

## Discussion

We conducted a comparative investigation of risk willingness in patients with MSA and PD considering participation in a clinical trial for disease-modifying therapy. Our analysis revealed similar perceptions of severity for hypothetical medication and surgical side effects in both patient groups, with mental drug side effects such as memory loss and personality changes perceived as most severe. Our initial hypothesis was a generally higher risk tolerance in the MSA cohort. However, interindividual willingness to accept therapy-associated risks varied widely for most scenarios. This finding contradicted our initial hypothesis. A considerable proportion of MSA patients, despite their substantially impaired quality of life, were not willing to accept increased therapy-associated risks. At group level, MSA patients displayed a non-significant trend towards higher willingness to accept therapy-associated risks than PD patients. Notably, we identified markedly different features associated with risk willingness between the two patient groups.

The comparison between therapy-associated risk decision making of the two patient groups is of particular interest for at least two reasons. First, MSA, and PD present with a similar clinical picture at disease onset. The reality, however, patients with MSA versus patients with PD face, is fundamentally different: MSA patients know they suffer from a fast-progressing deadly disease with no effective symptomatic therapy available, whereas effective pharmacotherapy and/or functional neurosurgery allows PD patients to survive with the disease in relatively good QoL for decades.

Second, and of general relevance, patient centricity receives increasing attention from physicians and regulatory agencies. Evaluations of patient motivation and risk tolerance must be conducted in a systematic, impartial, and evidence-based manner. Only then patients’ risk willingness can play a key role in physician consultations and shared decision-making. Likewise, this information allows to evaluate the impact of safety data in the design and conduct of clinical trials and provides guidance how to assess and weight different apparent side effects (risk benefit consideration) in the subsequent regulatory approval process.

Respective studies in chronic neurological disorders and clinical medicine as a whole are surprisingly sparse: Three independent studies investigated patients with multiple sclerosis. These patients would accept a median risk of sudden death of 1:10,000, but about 20% would not take any risk related to hypothetical disease-modifying therapies^11–13^.

In other medical disciplines studies on melanoma^14,15^—addressing patient preferences and treatment choices—have been conducted to evaluate patients’ preferences (“utilities”) for health states associated with interferon therapy. In irritable bowel syndrome (IBS) a study revealed a median accepted risk for hypothetical disease-modifying drugs. These patients would accept a median 1% risk of sudden death to cure their IBS symptoms for a 99% chance of cure^16^. In the context of aortic valve replacement^17^ and peripheral arterial disease^18^, death as a surgery complication was assessed, and the median risk acceptance for mortality from a curative medication was found to be up to 1%.

Our study has unique new features: first, we differentially assess, and compare risk-taking behavior of two different groups of patients in the same study. Second, we provide analyses at individual-level. MSA patients demonstrated a remarkable willingness, as indicated by their acceptance of a median 1% risk of sudden death for a median 99% probability of symptom cure. This median level is comparable to previous studies in patients suffering from IBS—a non-lethal disorder—and cardiovascular diseases (including lethal disorders), but much higher as in patients with multiple sclerosis^11–13^. PD patients reported a median 0.055% risk of sudden death, lower—though not significantly—on a group level than MSA patients. Our study has shown that analyses of therapy-associated risk willingness at group level cannot be representative of the whole patient population. Considering the lower quartile, a notable proportion of MSA (as well as PD) patients, despite their substantially impaired quality of life, were not willing to accept increased therapy-associated risks. This finding suggests that the attitude among many MSA patients is not one of “what can I lose”, but rather “I don’t want to lose even more”. Simultaneously, the upper quartile of MSA patients even accepted a risk of 25% or more sudden death for a 75% or less chance of cure compared to only a risk of 5% or more sudden death for a 95% or less chance of cure in PD. This extensive variability between individuals following a non-gaussian distribution and the lack of difference between MSA and PD at group level may have implications for the weighting of safety aspects in regulatory approval processes. There is no “one size fits all” solution.

Our findings underscore the necessity of a patient-centered approach in therapy development for MSA and PD. A simple inference from the disease (stage) to therapy-associated risk willingness is not feasible. Satisfaction with life situation, QoL nonmotor, and emotional subscores were associated with MSA, but not PD patients’ therapy-associated risk willingness. These aspects of psychological and nonmotor disease burden, significantly more pronounced in MSA patients in our cohort, appeared relevant to therapy-associated risk willingness solely in MSA. The importance of required social support as an associated feature for MSA patients could be interpreted in light of their higher disease burden compared to PD patients, necessitating greater assistance from relatives, and caregivers. MSA patients requiring high degrees of social support were less likely to accept therapy-associated risks, which may express the desire not to burden one’s family further due to additional side effects of therapy. Risk decision-making is a complex process, encompassing more than just the combination of clinical and psychosocial features, which explained 51.97% of the variance in median accepted risk of drug side effects for MSA patients and 25.29% for PD patients. Other personal attributes, such as risk aversion^19^, information presentation^20^, and comprehension, emotions, and prior healthcare experiences may also contribute.

There are several limitations of the study that should be addressed before the SG can be applied in the assessment of MSA and PD patients. First, the results of this study reflect a predominantly Caucasian population in Germany. The setting of a tertiary center may have resulted in more highly motivated patients than those not referred. Therefore, the results may not be generalizable to all MSA and PD populations. The data analysis focused on patients meeting diagnostic criteria, which may have led to an overrepresentation of advanced MSA stages. Due to the rarity of MSA, our cohort size was limited. Although we established a network of several university medical centers in Germany to yield a cohort of 49 MSA patients, future studies are needed to confirm these results using a larger sample size. Furthermore, patients were asked about the use of a hypothetical medication or surgery to cure their symptoms. When MSA and PD patients are confronted with a decision about a real medication or surgery—dependant on the level of familiarity with the offered intervention, their responses might be different. We did not explicitly assess the impact of cultural or religious beliefs on patient responses. The inability to control for medication fluctuations due to individual variability, the extended duration required to complete questionnaires (1.5–2 h), and the presence of participants without any dopaminergic medication (30 MSA patients and PD patients under DBS) is a significant limitation of our study. We did not specifically collect data on the participants’ history of occupation, including whether they were healthcare workers or held positions that might confer a greater understanding of medical research and risk assessment. Finally, we did not employ a specific rating scale for depression and anxiety. Presence of clinical depression was rated by a physician and incorporated as a categorical feature in our modeling so that the association with specific risk-willingness may have been underestimated.

In conclusion, we conducted a comprehensive study in α-synucleinopathies to quantify and compare MSA and PD patients’ risk willingness regarding drug side effects and surgery complications of varying intensity. Our study highlighted that, unexpectedly, individuals’ specific risk willingness followed a non-gaussian distribution. Knowledge and awareness of psychosocial and clinical features, such as degree of required social support, generalized risk-taking attitude, nonmotor or emotional disease burden may help to explain how individuals arrive at different therapy-associated risk decisions—with some individuals even accepting either no risks at all or remarkably high risks. These insights are crucial for physicians as they engage in shared decision-making with MSA and PD patients, helping to tailor discussions to the unique preferences and risk tolerances of each patient. By understanding these factors, physicians can better communicate the potential risks and benefits of medical procedures, thereby enhancing patient understanding and satisfaction with their care choices. Furthermore, the findings from this study could inform the design of future clinical trials and regulatory evaluations of new therapies for MSA and PD. By incorporating patient preferences into the early stages of therapy development and approval processes, researchers, and regulators can ensure that these therapies align more closely with patient needs and values. This approach could improve patient outcomes and enhance recruitment and retention in clinical studies, particularly in conditions with limited patient populations. However, the generalizability of our study is limited by its small, predominantly Caucasian sample from tertiary care centers in Germany and the use of hypothetical scenarios. Future research should explore these findings across diverse geographical and ethnic groups, including collaborations with primary care settings to compare real-world treatment outcomes with our hypothetical scenarios, providing actionable data for clinical practice.

## Methods

### Study design

We performed a multi-center, cross-sectional study at nine university and university-associated medical centers across Germany. Approvals from the respective ethical review boards were obtained (project number 147/47 central ethics committee Marburg). Eligible participants met consensus criteria for possible or probable MSA^21^ or MDS diagnostic criteria for the clinical diagnosis of PD^22^, respectively. Participants with cognitive impairment as measured by Montreal Cognitive Assessment score value below 24 were not enrolled due to potential reliability concerns in reporting health-related preferences^23^.

### Patient enrollment

After informed consent was given, participants were handed out a paper-pencil questionnaire to fill out in the outpatient setting, or, if this was ill-timed for any reason, at home, and sent back by surface mail. Participants were trained by a study physician to assess SGs, using a specific example scenario as a guide. Between March 2019 and March 2021, 49 patients with possible or probable MSA^21^, and 38 patients with PD^22^ were recruited by a network of nine medical centers in Germany with specialized movement disorders outpatient clinics.

### Risk willingness assessment

**Fig. 4:**
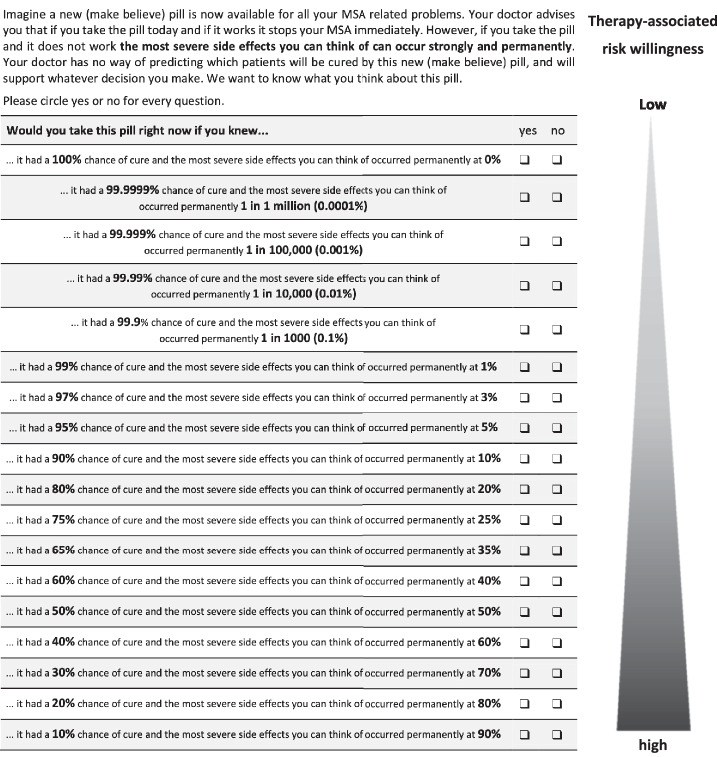
Standard gamble scenario for measuring therapy-associated risk willingness. Participants had to make direct explicit trade-offs between decreasing probabilities of being cured and increasing probabilities of side effects (answer yes or no for 18 response options at different percentages, e.g., “… it had a 100% chance of cure and the most bearable side effects you can think of occurred permanently at 0%”).

Specific therapy-associated risk willingness was measured by a set of six SGs. The participants were initially presented with a comprehensive list of potential side effects and complications that might arise from medication or surgical procedures. They were then instructed to select the top three mental and physical side effects that they could bear and those that were most severe. In the second stage of the process, the participants were required to provide a percentage estimate of the risks they were willing to take if given a drug or undergoing surgery. The potential outcomes of drug-related risks and surgery were either complete cure, permanent suffering from the previously selected bearable and severe side effects, or sudden death. As illustrated in Fig. 4, the SG method required participants to balance decreasing cure probabilities against increasing side effect probabilities. The primary endpoint of our study was the maximum accepted percent probability of side effects as a proxy for specific therapy-associated risk willingness.

To assess generalized risk-taking attitude, we used the RPS—a sum score of a seven-items scale of an individual’s general propensity to take risks with answers given on a six-point Likert scale (1 = not at all true to 6 = true)^11,24^. Furthermore, patients completed a 28-item survey on their views on the design of clinical trials, reasons for, and barriers against participation. Two items assessed required social support regarding clinical trial participation and were averaged to create a subscale. These were rated on a 6-point Likert scale from 1 (not at all true) to 6 (true) and included “transport options in my family are lacking” and “… others, e.g., family or friends do not support me.” The complete set of questionnaires is shown in Supplementary File 1. Additional descriptions of the measures and supplementary methods are provided in Supplement 1–4.

#### Quality of life assessment

To ensure direct comparability and minimize potential measurement bias, we administered the validated 40-item MSA quality of life (QoL) questionnaire^25^ to both MSA and PD patients. It assesses patients’ disease burden on a five-point Likert scale (0 = no problem to 4 = extreme problem). Motor, emotional, and nonmotor QoL subscores were calculated^25^ by summing items and, transforming to a range of 0–100 (100 × [(observed score minus minimum possible score)/(maximum possible score minus minimum possible score)]). For ease of interpretation, we inverted the resulting subscores so that high subscore values correspond to a high degree of QoL and low disease burden. We used these patient-reported motor, emotional, and nonmotor QoL subscores to explore correlations with therapy-associated risk willingness, highlighting the nuanced insights these patient-centered metrics provide over traditional clinical scores.

#### Disease-specific assessments

All patients were examined by board-certified neurologists who collected clinical and demographic data alongside this study (Supplementary Table 1 for detailed description). Disease severity was rated by validated scales (MDS-UPDRS^26^, UMSARS^27^, and Hoehn and Yahr^28^ staging) in the off-state (12 h without medication).

### Statistical analysis

The study was exploratory. Due to the rarity of MSA, our sample size was limited by the number of MSA patients that could be recruited by the nine university medical centers. Statistical analyses were performed in “R”, version 4.1.1^29^. We employed Wilcoxon rank-sum test to assess differences in therapy-associated risk willingness between patients with MSA and PD regarding the six SG scenarios. Categorical clinical and demographic variables of the study population as well as reasons and barriers towards clinical trial participation were analyzed and compared between subgroups using χ² tests. Numeric demographic and clinical variables were analyzed using Wilcoxon- or T-tests after checking for normality (Shapiro-Wilk test). All significance levels were set to *p* < 0.05. To control for false discovery rate in multiple comparisons, a Benjamini-Hochberg adjustment was applied where indicated. All tests were performed two-sided. Figures were produced using the package ggplot2 (version 3.4.2)^30^. For therapy-associated risk willingness regarding drug side effects, the median of three scenarios (most bearable, severe, and lethal side effects) was calculated and used for further analyses of associated factors. A similar procedure was applied regarding surgery complications. Bivariate correlations of continuous variables with the median of three scenarios were calculated using Pearson correlation coefficients. For ordinal scaled variables, Spearman correlation coefficients were calculated. Conditional variable importance in a multivariable model for the median of three scenarios was assessed using random forest regression^31^. We used standard parameters, grew 100 trees per run, and performed 200 runs to calculate 95% confidence intervals (“permimp” package, version 1.0.2). This approach effectively accommodates the high multicollinearity observed among associated features, providing unbiased, and stable results even for small sample sizes^32^.

### Supplementary information

Supplementary material

## Data Availability

The raw data used in preparation of the figures and tables will be shared in anonymized format upon reasonable request in agreement with EU legislation on the general data protection regulation, and be regulated in a material transfer agreement.
