# Supplementary material for: Risk willingness in multiple system atrophy and Parkinson’s disease understanding patient preferences
Source: NPJ Parkinsons Dis. 2024 Aug 15;10:158. doi: 10.1038/s41531-024-00764-5 (PMC11327309; doi:10.1038/s41531-024-00764-5)
Supplement: Supplementary file 1 — Supplementary material [file 41531_2024_764_MOESM1_ESM.pdf]

Supplementary material

Supplement 1. Study design

Participating centers

Movement Disorders Clinic, Beelitz-Heilstätten, Germany
Department of Neurology, TU Dresden, Dresden, Germany
Department of Neurology, University Medical Center Göttingen, Göttingen, Germany
Department of Neurology, Heidelberg University Hospital, Heidelberg, Germany
Department of Neurology, Jena University Hospital, Jena, Germany
Department of Neurology, University of Leipzig Medical Center, Leipzig, Germany
Department of Neurology, Philipps-University Marburg, Marburg, Germany
Department of Neurology with Institute of Translational Neurology, University of Muenster, Muenster, Germany
Department of Neurology, Ludwig-Maximilians-Universität München, Munich, Germany

Ethics and data protection

This study was conducted in accordance with the updated version of the international Council for Harmonisation of Good Clinical Practice. In addition, the study was performed according to the ethical principles outlined in the World Medical Association Declaration of Helsinki and its amendments. SecuTrial®, a database system that adheres to FDA regulations for clinical studies, was used for pseudonymized patient data entry.

Supplement 2. Survey development for MSA and PD patients

As we found no existing survey to assess MSA and PD patients' perception of, or willingness to accept, specific therapy-associated risks, we developed a 21-page survey based on well-stablished questionnaires [supplementary references 1-6] and adapted instruments to do so. We sought to assess patients' perceptions on different common *a priori* defined side effects of medication or surgery procedures. We then aimed to quantitatively determine patients' willingness to accept therapy-associated risks. The survey was piloted in a small convenience sample of patients with MSA (n = 3), and an equal number of PD (n = 3) and healthy control participants (n = 3). Adjustments were made to reflect solicited feedback by patients.

Supplement 3. Additional psychosocial measures

Reasons to participate and barriers towards participation were assessed with items adapted [supplementary reference 7] to the context of clinical trials against MSA. Answers were given on a 6-point Likert scale (1= not at all true to 6 = true) in response to eight items for reasons for participating ("I would take part in a clinical study only if ..., e.g., … the doctor would recommend me the trial"). Ten items were answered for reasons for declining participation ("I would not take part in a clinical study since…, e.g., … it involves a long or expensive journey"). Further reasons for participation and declining could be added by two open-answer fields each and rated on the same scale. Attitude towards participation was assessed with "For me, participation in a clinical trial against MSA would be…", followed by four 6-point semantic differentiation items (e.g. "beneficial/harmful") [supplementary reference 8]. Intention to participate in a clinical trial was assessed with two items (e.g. "I intend

to participate in a clinical study for people with MSA when I am invited to participate.") on a 6-point Likert scale (1= not at all true to 6 = true). Anxiety was assessed using two items from the MSA-QoL questionnaire regarding concerns about the future ("In the last 4 weeks have you worried about the future?") and family impact ("In the last 4 weeks have you worried about your family?"). These items were utilized to form an anxiety subscale, ranging from 0 (no anxiety) to 4 (maximum anxiety), to assess its effect on risk willingness.

Supplement 4. Supplementary statistical analyses

We used the Shapley Additive Explanations (SHAP) approach [supplementary reference 9] to show at an individual level how a specific feature value common to everyone (e.g., being a female or having a low satisfaction with life situation) was associated with specific therapy-associated risk willingness. In game theory, this approach assigns an importance value (i.e., SHAP) to each feature, determining a player's contribution to success. Shapley values are well recognised for providing both local and global interpretability for tree-based models [supplementary reference 10]. Analysis of Shapley values was performed in "R", version 4.1.1 using the "fastshap" package version 0.0.7.

Supplementary Figure 1. Reasons for and barriers against participation in clinical trials

Percentages of agreement to statements are shown. Stars indicate significant group differences (χ^2 tests, $p < 0.05$). The importance of the benefits of treatment outweighing side effects was found to be more relevant for MSA patients compared to PD patients in deciding to participate in clinical trials ('Benefits of treatment in the trial outweighed side effects': MSA: 86%, PD 63%, p=0.0415, χ^2 test). "Long and expensive journey required" was found to be more relevant for PD patients compared to MSA patients when considering declining participation (MSA: 57%, PD 78%, p=0.0392, χ^2 test).

Supplementary Figure 2. Patients' attitudes towards surgery related risks

Patients were presented with *a priori* defined lists of possible surgery complications that they would suffer from permanently. They were asked to tick the three most severe (a) or most bearable (b) complications. Percentages of chosen side effects are displayed. Both groups considered leg or arm paresis (MSA 84%, PD 97%), memory loss (MSA 80%, PD 71%) and epileptic seizures (MSA 59%, PD 66%) as most severe. Hearing loss (MSA 65%, PD 76%) and loss of taste (MSA 61%, PD 79%) were considered by both groups as most bearable. They differed in their rating of memory loss (MSA 6%, PD 26%, p=0.02, χ^2 test) and secondary bleeding (MSA 29%, PD 8%, p=0.03, χ^2 test) as most bearable surgery complications.

Supplementary Figure 3. Patients' willingness to take risks regarding severity of surgery complications

Individuals' answers to standard gamble (SG) scenarios are shown. Each dot represents one patient and his or her maximum accepted risk for the respective scenario. Horizontal lines show median and interquartile ranges. No significant group differences between MSA and PD regarding the 3 SG could be detected (Wilcoxon-tests). Compared to drug side effects, the quantitative evaluation of surgery complications showed an opposite, albeit non-significant, trend: patients had a lower tolerance for severe complications compared to death following surgery (death after surgery: MSA median: 1% [Q1–Q3: 0.001–20%] risk, PD median: 1% [Q1–Q3: 0.001–7.5%] risk vs. most severe surgery complications: MSA median: 1% [Q1–Q3: 0.001–20%] risk, PD median:

0.1% [Q1–Q3: 0.001–10%] risk). At group level, bearable surgery complications were tolerated to a greater extent than the severe or lethal surgery complications (MSA median: 4% [Q1–Q3: 0.01–32.5%] risk, PD median: 1% [Q1–Q3: 0.01–10%] risk). Comparing risk propensities for drug side effects vs. surgery complications revealed no significant within group differences, with the exception that PD patients accepted bearable surgery complications with a lower likelihood than bearable drug side effects (p=0.003, Wilcoxon).

Supplementary Figure 4. Spreads of individuals' risk tolerance towards drug side effects

MSA and PD patients' specific risk willingness regarding drugs were compared between most severe and lethal or most bearable and lethal side effects. Lines connect observations of the same individual. Green lines indicate decreasing accepted risk of sudden death as a side effect, black lines indicate increasing accepted risk of sudden death as a side effect. A considerable fraction of patients was more willing to accept sudden death than permanent severe drug side effects (25% of MSA and PD patients). This observation was less pronounced regarding bearable side effects: 12.5% of MSA and 16.7% of PD patients preferred sudden death over most bearable drug side effects.

Supplementary Figure 5. Spreads of individuals' risk tolerance towards surgery complications

MSA and PD patients' specific risk willingness regarding surgery were compared between most severe and lethal or most bearable and lethal complications. Lines connect observations of the same individual. Green lines indicate decreasing accepted risk of sudden death as a complication, black lines indicate increasing accepted risk of sudden death as a complication. A considerable fraction of patients was more willing to accept sudden death than permanent severe surgery complications (34.8% and 31.4% of MSA and PD patients). 15.2% of MSA and 31.4% of PD patients preferred sudden death over most bearable surgery complications.

Supplementary Figure 6. Variable importance of clinical and psychosocial features in association with risk decision making for investigational surgery procedures.

The x-axis displays the conditional variable importance values obtained from random forest regression analyses conducted separately for PD (blue) and MSA (red) patients. Shown are point estimates and 95% confidence intervals obtained from 200 runs. The variable with the highest importance is assigned a value of 100%, and all other variables are expressed as a percentage relative to that value. Any variables with confidence intervals that include zero or negative values are considered to have no predictive power in our model and are assigned a value of zero.

The model explained 31.59% of the variance in median accepted risk of drug side effects for MSA patients (R squared) and 30.15% for PD patients, as determined by 10-fold cross-validation. Compared to drug side effects, random forest regression yielded similar associated features regarding the median accepted risk of surgery complications. Degree of required social support ranked higher for MSA patients. 95% confidence intervals for age and sex reached zero, thus they could not be considered as associated features. For PD patients, sex, age, disease duration and quality of life motor subscore were associated with median accepted risk of surgery complications. PD patients with higher median accepted risk of surgery complications were more likely male, relatively young, suffered relatively long from the disease and displayed lower motor quality of life subscores. As opposed to median accepted risk of drug side effects, RPS was not among the associated features.

Supplementary Figure 7. Features associated with risk decision making for investigational drugs at individual level

SHAP values are shown on the x-axis. We conducted the analysis of SHAP values to show at individual level how a specific feature value (e.g., being a female or having a low satisfaction with life situation) was associated with specific therapy-associated risk willingness. This analysis was conducted for significant associated features identified through conditional variable importance metrics from random forest regression.

In **a)** and **e)**, each dot represents an individual with MSA or PD, respectively. The color of the dot indicates the value of a specific feature, with high values being represented by orange and low values by purple. For example, individuals with high satisfaction with their life situation (orange dots) displayed negative SHAP values, while those with low satisfaction displayed positive SHAP values. This means that high satisfaction was associated with low willingness to take therapy-associated risks, and vice versa. Sex is represented by recoded values, with low values (0) indicating male and higher values (1) indicating female.

In **b)-d)** three representative patients with MSA and in **e)-g)** three patients with PD are shown, the ones with the lowest and highest therapy-associated risk willingness, as well as one patient with medium therapy-associated risk willingness. Features that were associated with a higher therapy-associated risk willingness in an individual patient are displayed in red, whereas features that were associated with a lower therapy-associated risk willingness in an individual patient are displayed in blue. For example, the most important features of the MSA patient with the highest therapy-associated risk willingness displayed in **b)** were a relatively high risk propensity score (32), a medium quality of life nonmotor subscore (48) and a relatively low satisfaction with life situation (11). In contrast, the most important features of the MSA patient with the lowest therapy-associated risk willingness were a medium satisfaction with life situation (60), a relatively low risk propensity score (10) and a relatively high quality of life nonmotor subscore (83).

Supplementary Figure 8. Features associated with risk decision making for investigational surgery procedures at individual level

SHAP values are shown on the x-axis. We conducted the analysis of SHAP values to show at individual level how a specific feature value (e.g., being a female or having a low satisfaction with life situation) was associated with specific therapy-associated risk willingness. This analysis was conducted for significant associated features identified through conditional variable importance metrics from random forest regression.

In **a)** and **e)**, each dot represents an individual with MSA or PD, respectively. The color of the dot indicates the value of a specific feature, with high values being represented by orange and low values by purple. For example, individuals with high satisfaction with their life situation (orange dots) displayed negative SHAP values, while those with low satisfaction displayed positive SHAP values. This means that high satisfaction was associated with low willingness to take therapy-associated risks, and vice versa. Sex is represented by recoded values, with low values (0) indicating male and higher values (1) indicating female.

In **b)-d)** three representative patients with MSA and in **e)-g)** three patients with PD are shown, the ones with the lowest and highest therapy-associated risk willingness, as well as one patient with medium therapy-associated risk willingness. Features that were associated with a higher therapy-associated risk willingness in an individual patient are displayed in red, whereas features that were associated with a lower therapy-associated risk willingness in an individual patient are displayed in blue. For example, the most important features of the MSA patient with the highest therapy-associated risk willingness were a relatively high risk propensity score (32), a low degree of required social support (1) and a relatively low satisfaction with life situation (11). In contrast, the most important features of the MSA patient with the lowest therapy-associated risk willingness were a high degree of required social support (6), a medium satisfaction with life situation (60), a relatively low risk propensity score (10), and relatively high quality of life emotional (95) and nonmotor (83) subscores.

Supplementary Figure 9. Bivariate correlations of clinical and psychosocial features with patients' willingness to take drug related risks

Bivariate associations between clinical, psychosocial features, and the median accepted risk of drug side effects are presented. MSA and PD patients are represented as red triangles and blue dots, respectively. Pearson correlation coefficients ('r') were used to analyze continuous variables such as quality of life subscores. Point-biserial correlation ('r') was used for categorical variables, and Spearman correlation coefficients ('ρ') were employed for ordinal scaled variables like Hoehn and Yahr stage. Regression curves and 95% confidence intervals are provided for continuous variables. P-values ('p'), both raw and adjusted following the Benjamini-Hochberg procedure, were computed (raw p-values are shown in brackets). In supplementary Figure 9 h), values of 5 on the x axis represent a positive attitude, lower values represent an increasingly negative attitude towards clinical trials. In supplementary Figure 9 i), values of 5 on the x axis represent a high intention, lower values represent an increasingly lower intention to participate in clinical trials. In supplementary Figure 9 j), values of 4 on the x axis represent maximum anxiety, lower values represent lower degrees of anxiety. LDD= Levodopa daily dose in mg.

Supplementary Figure 10. Bivariate correlations of clinical and psychosocial features with patients' willingness to take surgery related risks

Bivariate associations between clinical, psychosocial features, and the median accepted risk of drug side effects are presented. MSA and PD patients are represented as red triangles and blue dots, respectively. Pearson correlation coefficients ('r') were used to analyze continuous variables such as age, disease duration, quality of life subscores, and RPS. Point-biserial correlation ('r') was used for categorical variables, and Spearman correlation coefficients ('ρ') were employed for ordinal scaled variables like the degree of required social support or Hoehn and Yahr stage. Regression curves and 95% confidence intervals are provided for continuous variables. P-values ('p'), both raw and adjusted following the Benjamini-Hochberg procedure, were computed (raw p-values are shown in brackets). In supplementary Figure 10 p), values of 5 on the x axis represent a positive attitude, lower values represent an increasingly negative attitude towards clinical trials. In supplementary Figure 10 q), values of 5 on the x axis represent a high intention, lower values represent an increasingly lower intention to participate in clinical trials. In supplementary Figure 10 r), values of 4 on the x

axis represent maximum anxiety, lower values represent lower degrees of anxiety. LDD= Levodopa daily dose in mg.

Supplementary Figure 11. MSA patients' willingness to take risks regarding severity of drug or surgery side effects

Individuals' answers to standard gamble (SG) scenarios are shown. Each triangle represents one patient and his or her maximum accepted risk for the respective scenario. There is a trend to accept less specific therapy-associated risks among MSA-C patients (Wilcoxon-tests, p-value = 0.061 regarding median accpeted risk drugs, p-value = 0.15 regarding median accepted risk surgery).

Supplementary file 1.

Questionnaire including paper standard gamble scenarios distributed to MSA patients (a similar version was given to PD patients with the word "MSA" replaced by "PD"). Questionnaires were originally distributed in German and translated into English for this publication.

Supplementary file 2.

STrengthening the Reporting of OBservational studies in Epidemiology (STROBE) statement

Supplementary References

[1] Green C, Brazier J, Deverill M. Valuing health-related quality of life. A review of health state valuation techniques. Pharmacoeconomics 2000;17:151–65.

[2] Gafni A. The standard gamble method: what is being measured and how it is interpreted. Health Services Research 1994;29:207.

[3] Torrance GW, Feeny DH, Furlong WJ, Barr RD, Zhang Y, Wang Q. Multiattribute utility function for a comprehensive health status classification system. Health Utilities Index Mark 2. Med Care 1996;34:702–22.

[4] Meertens RM, Lion R. Measuring an Individual's Tendency to Take Risks: The Risk Propensity Scale1. Journal of Applied Social Psychology 2008;38:1506–20. https://doi.org/10.1111/j.1559-1816.2008.00357.x.

[5] Glanz BI, Greeke E, LaRussa A, Stuart F, Rintell DJ, Chitnis T, et al. Risk attitudes and risk perceptions in individuals with multiple sclerosis. Multiple Sclerosis Journal–Experimental, Translational and Clinical 2016;2:2055217316665406.

[6] Schrag A, Geser F, Stampfer-Kountchev M, Seppi K, Sawires M, Köllensperger M, et al. Health-related quality of life in multiple system atrophy. Movement Disorders 2006;21:809–15. https://doi.org/10.1002/mds.20808.

[7] Jenkins V, Fallowfield L. Reasons for accepting or declining to participate in randomized clinical trials for cancer therapy. British Journal of Cancer. 2000;82(11):1783-1788. doi:10.1054/bjoc.2000.1142.

[8] Ajzen I. The theory of planned behavior. Organ Behav Hum Decis Process. 1991;50(2):179-211. doi:10.1016/0749-5978(91)90020-T.

[9] Lundberg, S. M., & Lee, S. I. (2020). A unified approach to interpreting model predictions. In Advances in neural information processing systems (pp. 4765-4774).

[10] Lundberg, S. M., Erion, G. G., Chen, H., DeGrave, A., Prutkin, J. M., Nair, B., et al. (2020). From local explanations to global understanding with explainable AI for trees. Nature Machine Intelligence, 2(1), 56-67.

Supplementary table 1. Clinical and demographic characteristics of the study cohort

	Overall		MSA overall		MSA-P		MSA-C		PD		p (MSA-P vs MSA-C)	p (MSA vs PD)
N (%)	87	(100.0)	49	(100.0)	28	(56.9)	21	(43.1)	38	(100.0)		
Diagnostic certainty												
Possible, n (%)			10	(20.4)	4	(14.3)	6	(28.6)				
Probable, n (%)			39	(79.6)	24	(85.7)	15	(71.4)				
Sex											0.196	0.112
Women, n (%)	38	(43.7)	25	(51.0)	11	(39.3)	13	(61.9)	14	(36.8)		
Men, n (%)	49	(56.3)	24	(49.0)	17	(60.7)	8	(38.1)	24	(63.2)		
Age												
Age at study entry, mean (SD)	65.0	(9.8)	63.7	(8.8)	65.3	(8.4)	61.7	(9.0)	66.5	(10.7)	0.165	0.187
Age at primary diagnosis, mean (SD)	60.4	(10.7)	61.8	(8.9)	63.6	(8.3)	59.5	(9.2)	58.5	(12.4)	0.1242	0.158
Time since primary diagnosis in years, mean (SD)	4.8	(4.9)	2.1	(1.7)	1.9	(1.6)	2.3	(1.8)	8.3	(5.5)	0.389	**> 0.001**
Hoehn and Yahr stage, mean (SD)	3.2	(1.0)	3.61	(0.9)	3.76	(0.9)	3.4	(1.0)	2.7	(0.8)	0.168	**> 0.001**
Stage 1, n (%)	2	(2.3)	0	(0.0)	0	(0.0)	0	(0.0)	2	(5.3)		
Stage 2, n (%)	19	(21.8)	4	(8.2)	1	(3.6)	3	(14.3)	15	(39.5)		
Stage 3, n (%)	40	(46.0)	23	(46.9)	12	(42.9)	11	(52.4)	17	(44.7)		
Stage 4, n (%)	15	(19.5)	12	(24.5)	9	(32.1)	3	(14.3)	3	(7.8)		
Stage 5, n (%)	11	(12.6)	10	(20.4)	6	(21.4)	4	(19.0)	1	(2.6)		
Scales												
UPDRS I mean (SD)	2.7	(2.0)	2.7	(2.6)					2.7	(2.1)		0.818
UPDRS II mean (SD)	14.3	(7.5)	24.0	(8.9)					11.8	(4.6)		**> 0.001**
UPDRS III mean (SD)	30.4	(14.0)	43.6	(12.2)	45.5	(11.4)	38.3	(13.9)	23.3	(9.0)	0.292	**> 0.001**
UMSARS I mean (SD)			20.3	(6.8)	16.8	(4.5)	23.2	(7.3)				
UMSARS II mean (SD)			21.6	(7.6)	18.2	(6.3)	24.5	(7.9)				
Clinical Symptoms												
Gait freezing, n (%)	10	(11.5)	5	(10.2)	3	(10.7)	2	(9.5)	5	(13.2)	1	1
Bradykinesia, n (%)	79	(90.8)	40	(81.6)	29	(103.6)	12	(57.1)	39	(102.6)	**0.004**	0.181
Rest tremor, n (%)	35	(40.2)	14	(28.6)	9	(32.1)	5	(23.8)	21	(55.3)	0.83	0.054
Postural Tremor, n (%)	9	(10.3)	6	(12.2)	4	(14.3)	2	(9.5)	3	(7.9)	1	0.664
Rigidity, n (%)	69	(79.3)	35	(71.4)	25	(89.3)	10	(47.6)	34	(89.5)	**0.007**	0.364
Postural Instability, n (%)	42	(48.3)	30	(61.2)	23	(82.1)	7	(33.3)	12	(31.6)	**0.002**	**0.005**
Depression, n (%)	30	(34.5)	17	(34.7)	10	(35.7)	7	(33.3)	13	(34.2)	1	0.921
Constipation, n (%)	42	(48.3)	14	(28.6)	10	(35.7)	4	(19.0)	28	(73.7)	0.390	**> 0.001**
Dystonia, n (%)	10	(11.5)	6	(12.2)	5	(17.9)	1	(4.8)	4	(10.5)	0.376	1
Autonomic failure, n (%)	66	(75.9)	42	(85.7)	25	(89.3)	17	(81.0)	24	(63.2)	1	**> 0.001**
Urinary incontinence, n (%)	50	(57.5)	34	(69.4)	20	(71.4)	14	(66.7)	16	(42.1)	1	**> 0.001**
Incomplete bladder emptying, n (%)	28	(32.2)	24	(49.0)	17	(60.7)	7	(33.3)	4	(10.5)	0.222	**> 0.001**
Impotence, n (%)	25	(28.7)	17	(34.7)	12	(42.9)	5	(23.8)	8	(21.1)	0.332	0.168
Orthostatic hypotension, n (%)	45	(51.7)	32	(65.3)	19	(67.9)	13	(61.9)	13	(34.2)	1	**0.003**
Cerebellar involvement, n (%)	32	(36.8)	31	(63.3)	14	(50.0)	22	(104.8)	1	(2.6)	**0.015**	**> 0.001**
Gait ataxia, n (%)	31	(35.6)	30	(61.2)	13	(46.4)	17	(81.0)	1	(2.6)	**0.008**	**> 0.001**
Limb ataxia, n (%)	25	(28.7)	24	(49.0)	9	(32.1)	15	(71.4)	1	(2.6)	**0.003**	**> 0.001**
Ataxic dysarthria, n (%)	21	(24.1)	21	(42.9)	5	(17.9)	16	(76.2)	0	(0.0)	**> 0.001**	**> 0.001**
Cerebellar oculomotor dysfunction, n (%)	14	(16.1)	14	(28.6)	1	(3.6)	13	(61.9)	0	(0.0)	**> 0.001**	**> 0.001**
Pyramidal involvement, n (%)	16	(18.4)	16	(32.7)	11	(39.3)	5	(23.8)	0	(0.0)	0.468	**>0.001**
Babinski sign positive, n (%)	6	(6.9)	6	(12.2)	4	(14.3)	2	(9.5)	0	(0.0)	1	0.057
Hyperreflexia, n (%)	12	(13.8)	12	(24.5)	8	(28.6)	4	(19.0)	0	(0.0)	0.735	**0.002**
Treatment												
Levodopa treatment, n (%)	65	(74.7)	27	(55.1)	22	(78.6)	5	(23.8)	38	(100.0)	0.478	0.845
Dopamine agonist treatment, n (%)	21	(24.1)	1	(2.0)	1	(3.6)	0	(0.0)	20	(52.6)	0.648	**> 0.001**
Levodopa response											0.173	**> 0.001**
Beneficial response, n (%)	51	(58.6)	13	(26.5)	10	(35.7)	3	(14.3)	38	(100.0)		
No or poor response, n (%)	28	(32.2)	28	(57.1)	15	(53.6)	13	(61.9)	0	(2.3)		
Unknown, n (%)	8	(9.2)	8	(16.3)	3	(10.7)	5	(23.8)	0	(0.0)		
Deep brain stimulation, n (%)	9	(10.3)	2	(4.1)	0	(0.0)	2	(9.5)	7	(18.4)	0.551	**0.049**
Secondary education											0.450	0.668
≤12 years, n (%)	37	(42.5)	23	(46.9)	16	(57.1)	7	(33.3)	14	(36.8)		
>12 years, n (%)	49	(56.3)	26	(53.1)	12	(42.9)	14	(66.7)	23	(60.5)		
Unknown, n (%)	1	(1.1)	0	(0.0)	0	(0.0)	0	(4.5)	1	(2.6)		

Supplementary Figure 1. Reasons for and barriers against participation in clinical trials

(a) Percentages of agreement to reasons for participating in clinical trials

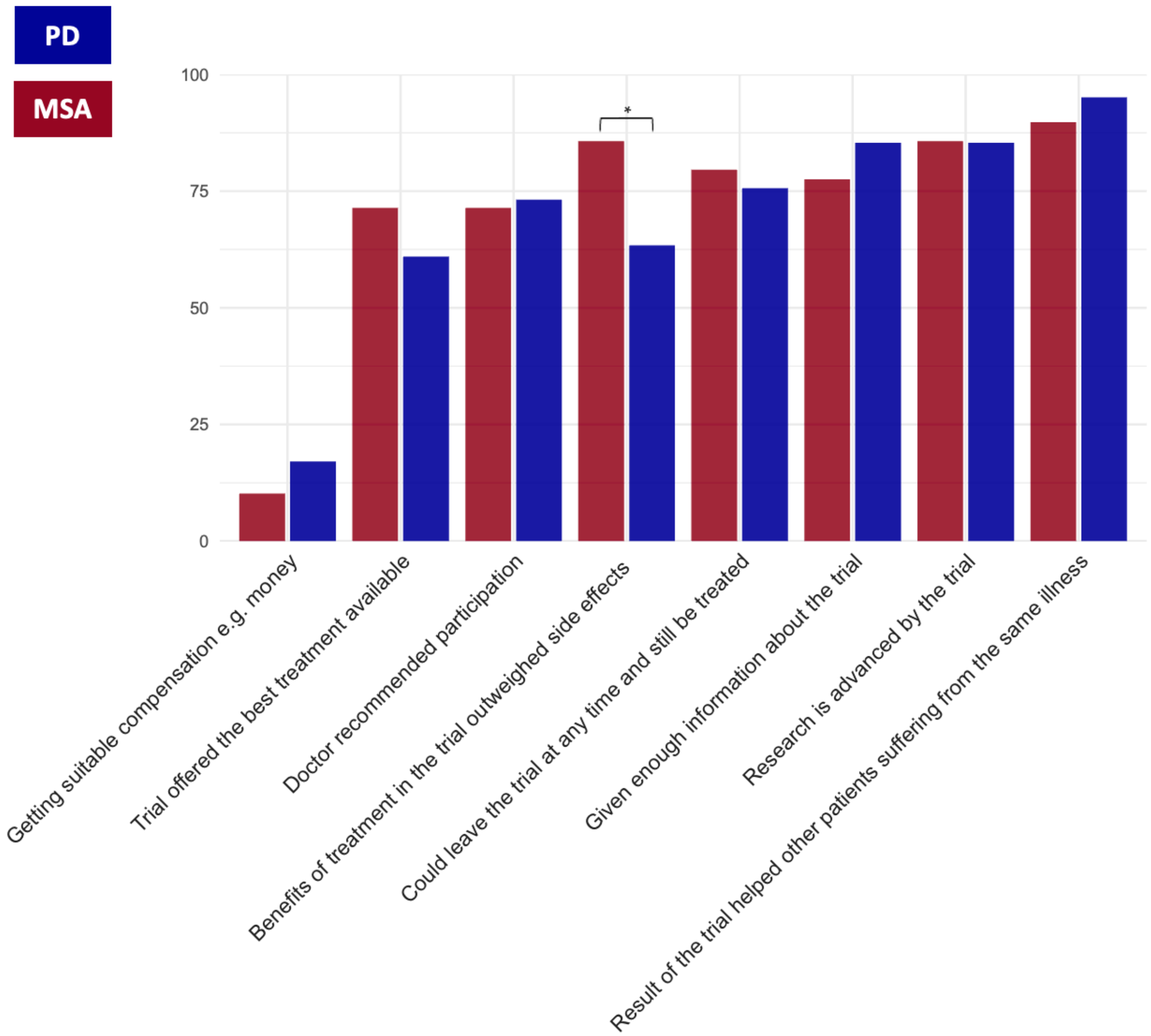

(b) Percentages of agreement to barriers against participation

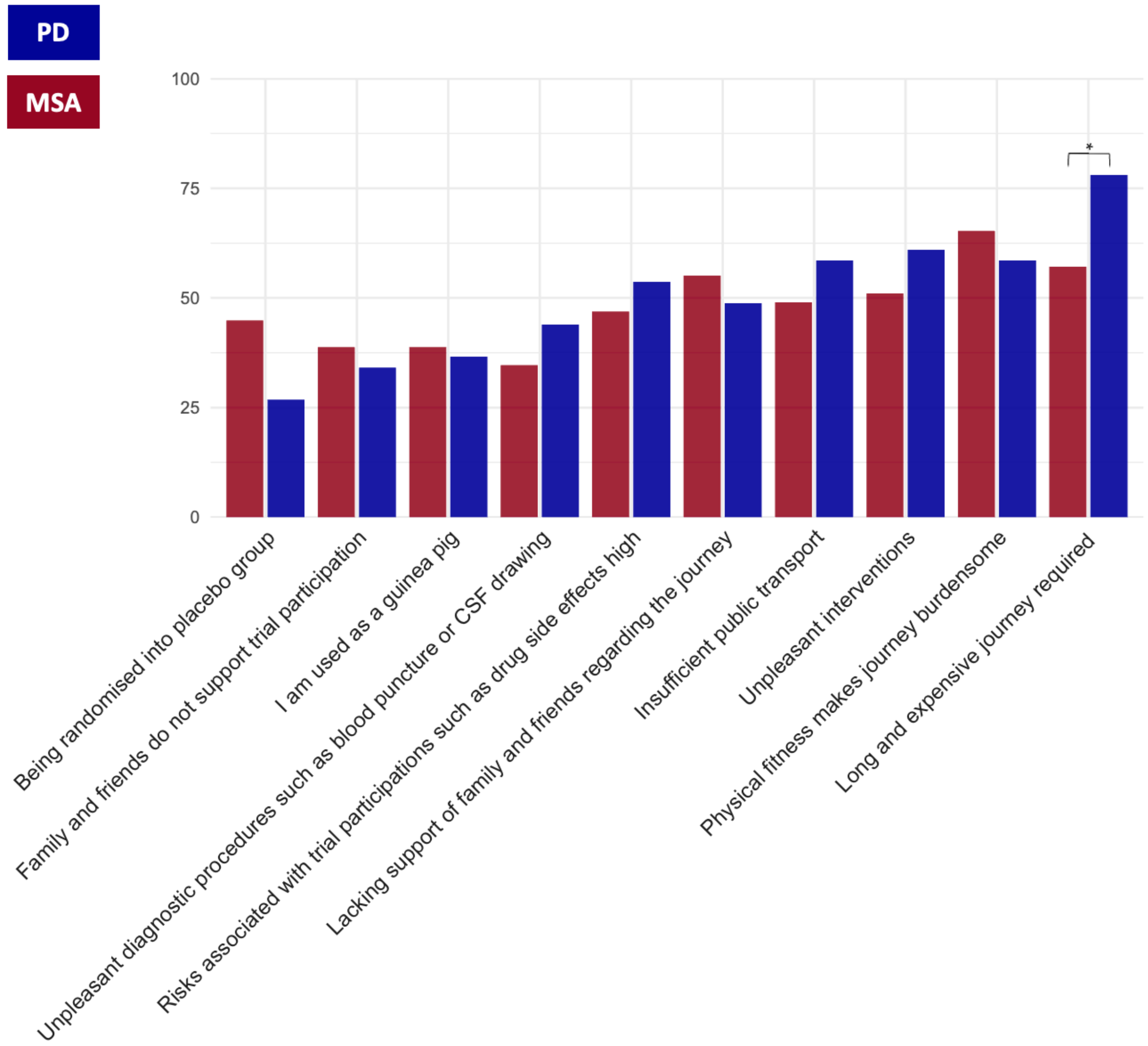

Supplementary Figure 2. Patients' attitudes towards surgery related risks

a) Percentages of chosen most **severe** surgery complications

b) Percentages of chosen most **bearable** surgery complications

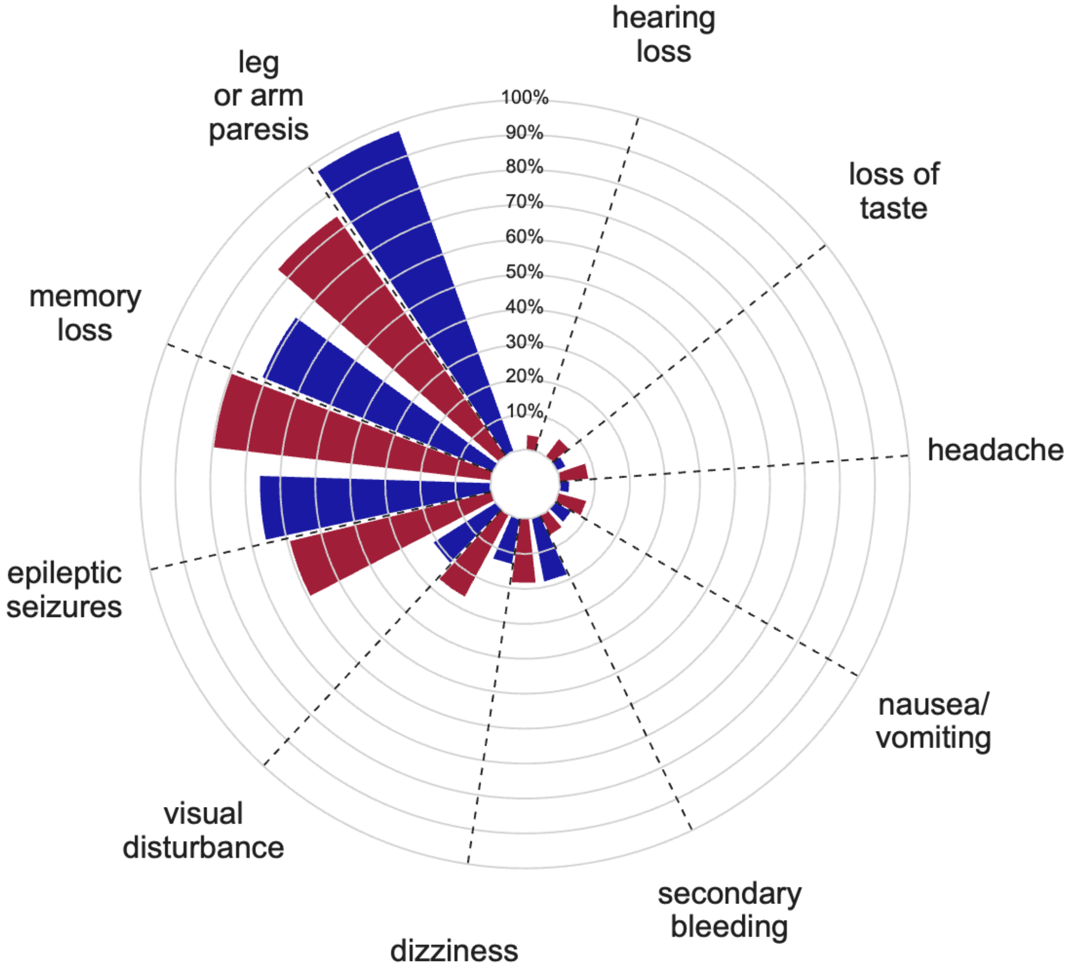

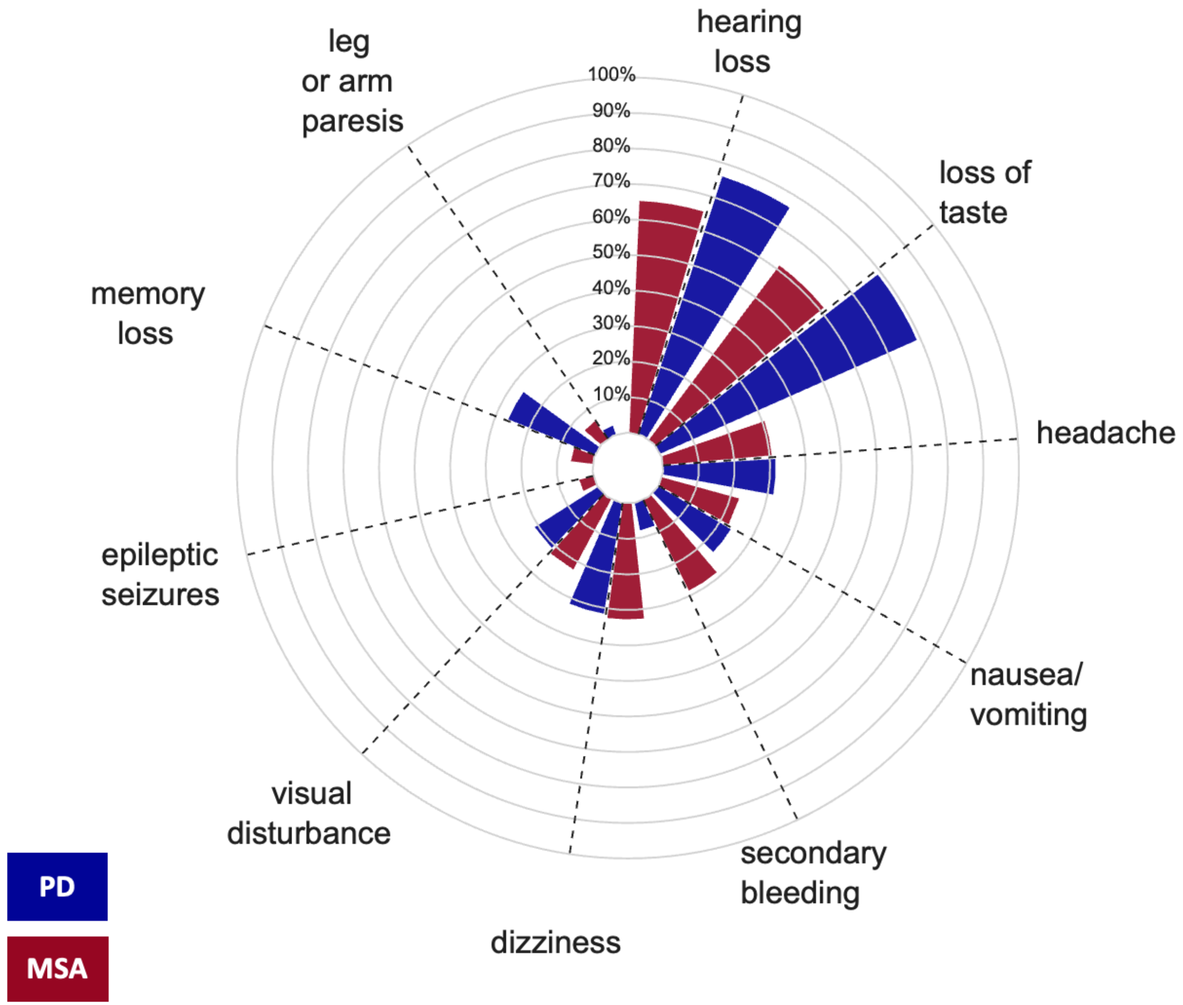

Supplementary Figure 3. Patients‘ willingness to take risks regarding severity of surgery complications

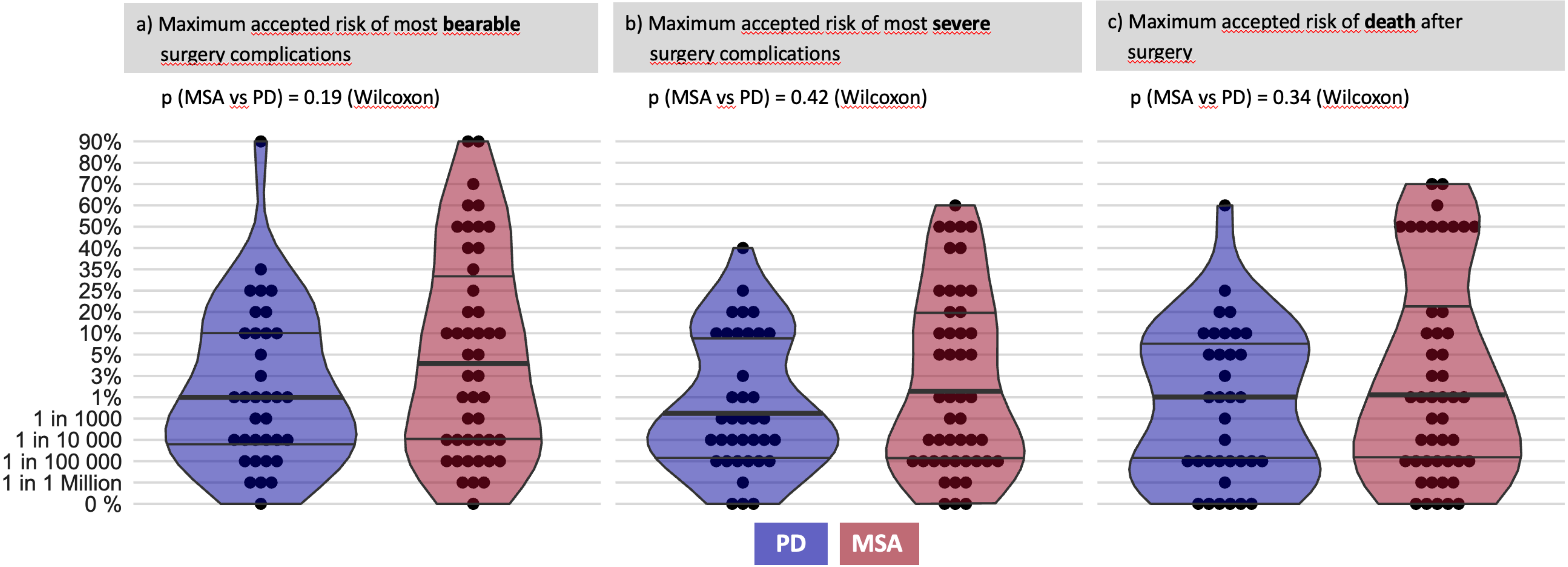

Supplementary Figure 4. Spreads of individuals' risk tolerance towards drug side effects

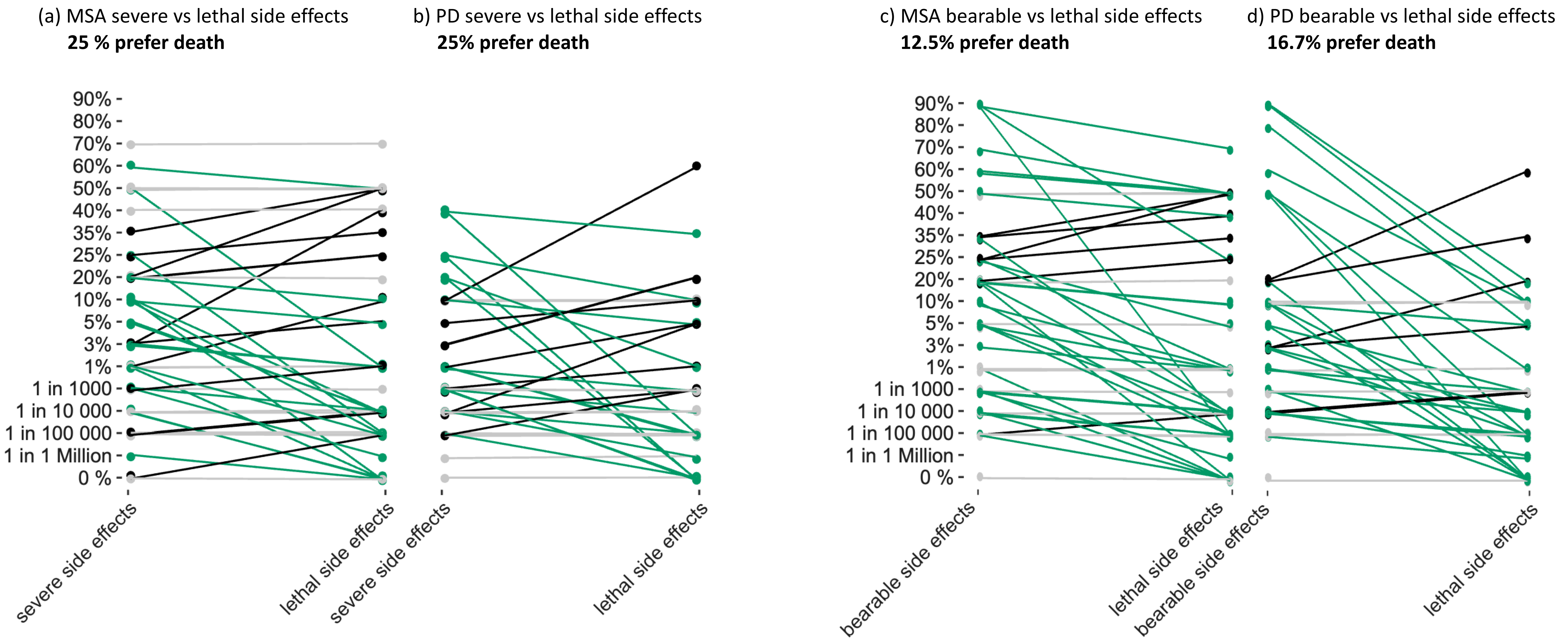

Supplementary Figure 5. Spreads of individuals' risk tolerance towards surgery complications

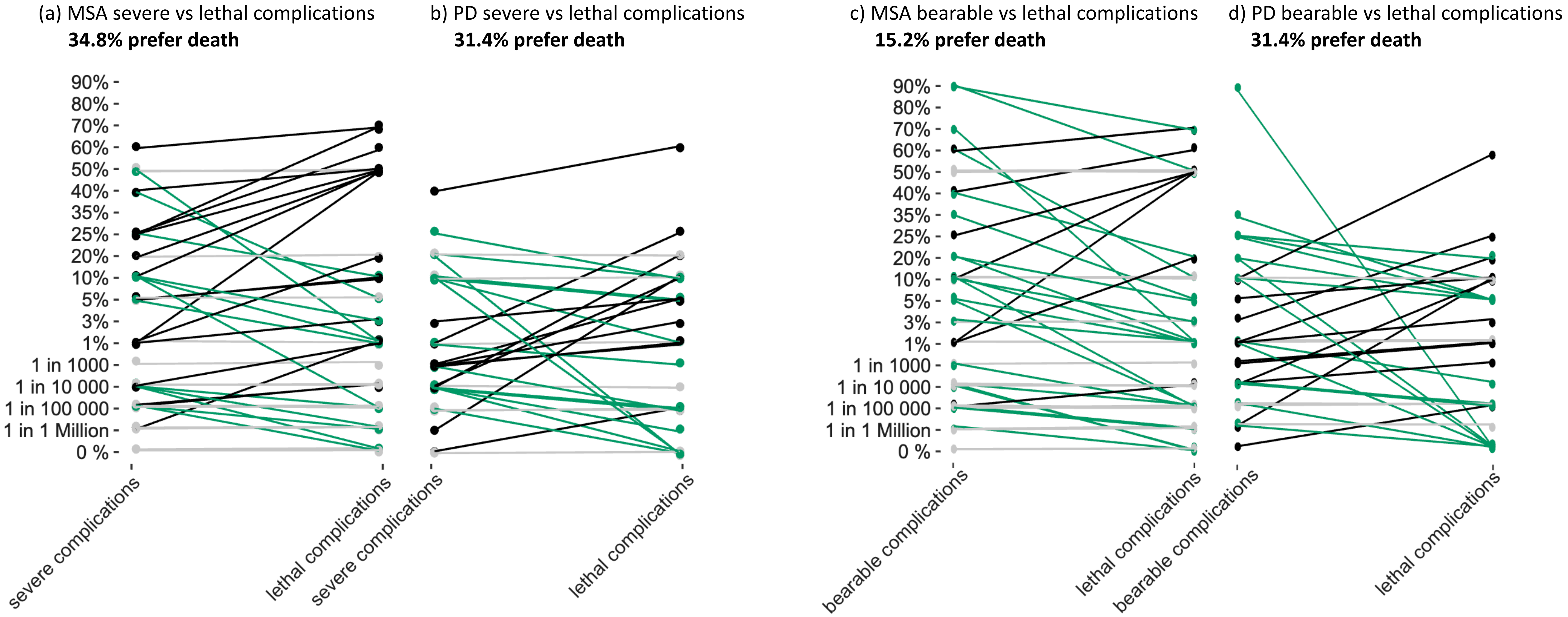

Supplementary Figure 6. Variable importance of clinical and psychosocial features in association with risk decision making for investigational surgery procedures

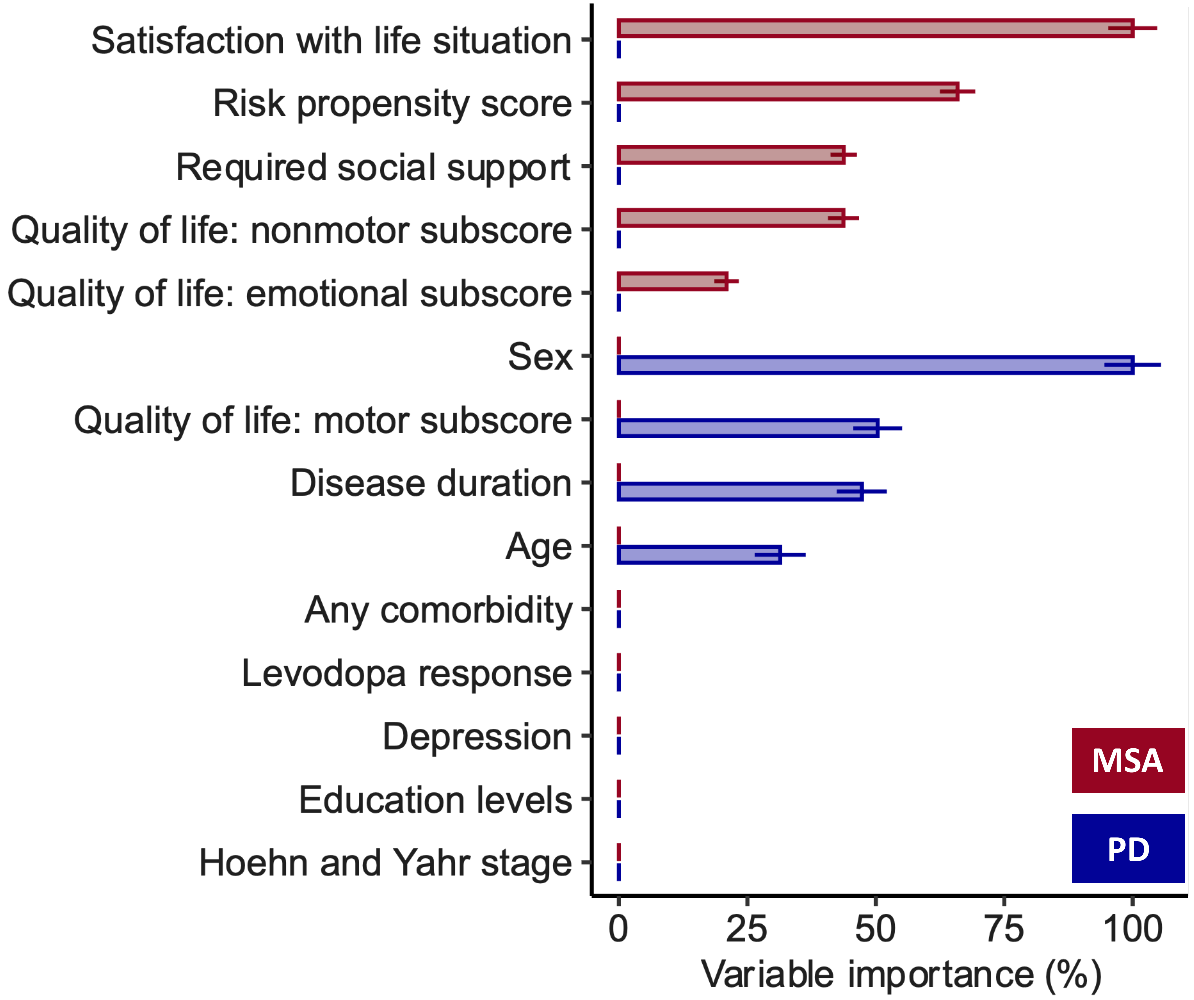

Supplementary Figure 7. Features associated with risk decision making for investigational drugs at individual level

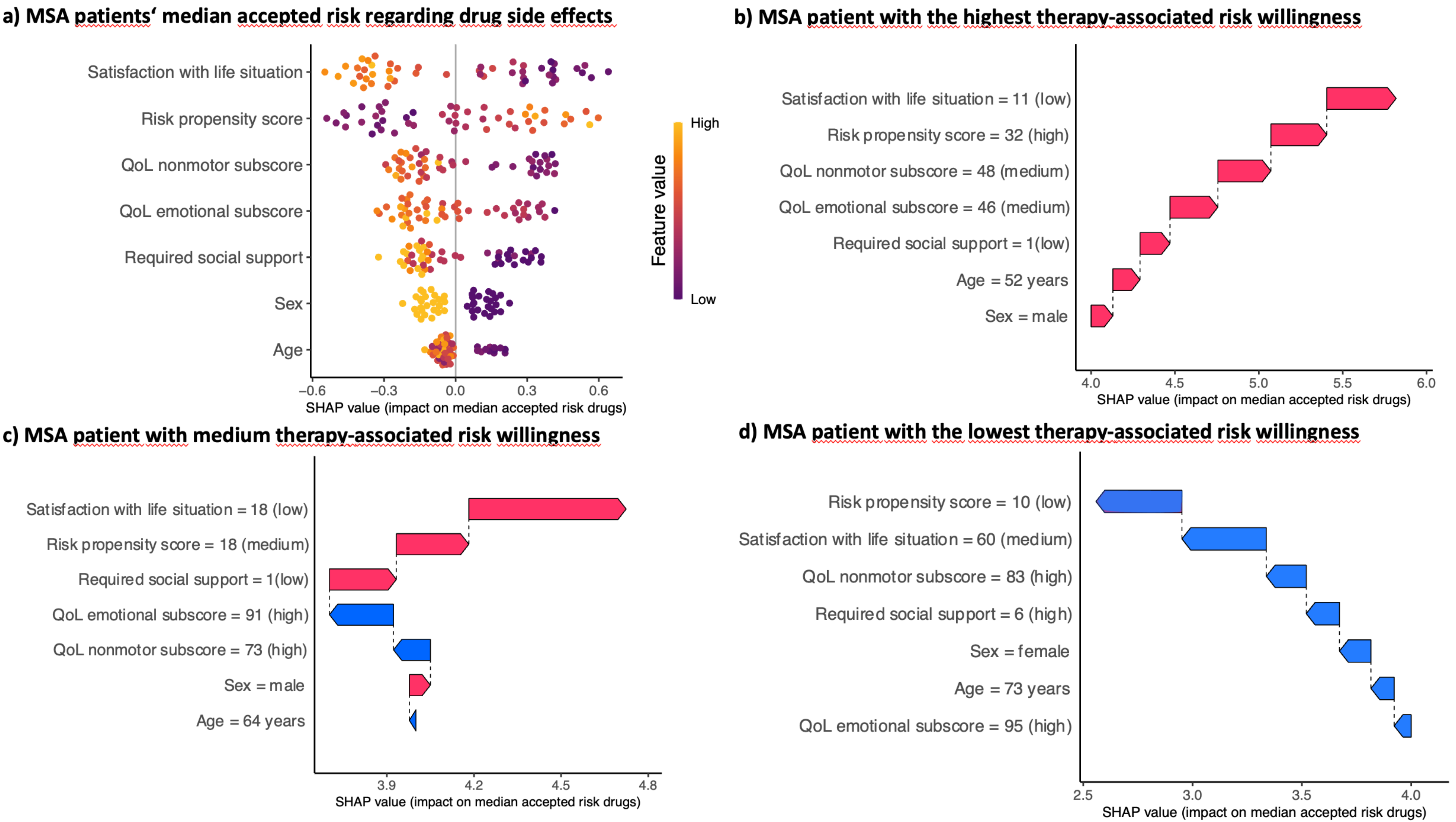

Supplementary Figure 7. Features associated with risk decision making for investigational drugs at individual level

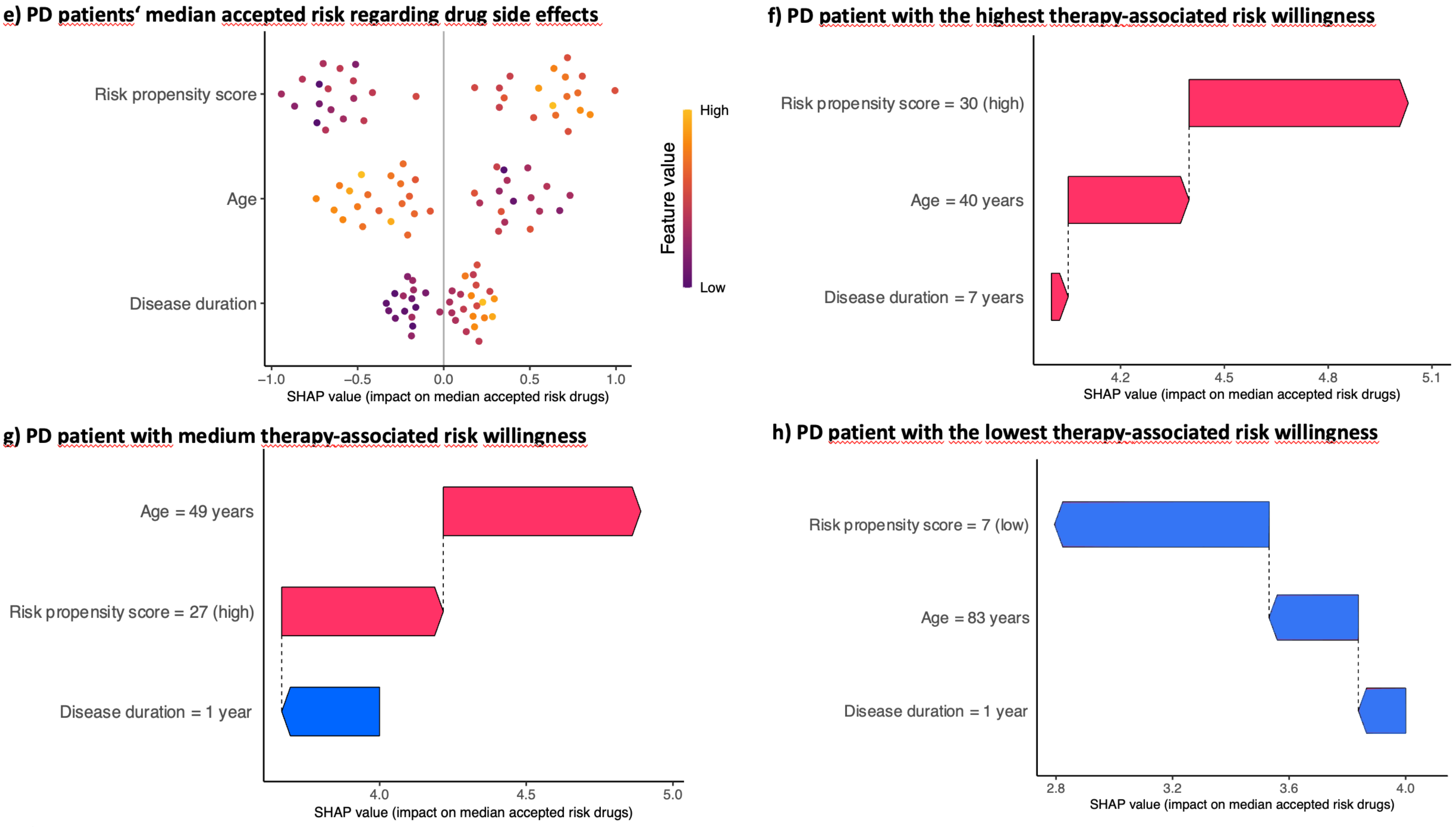

Supplementary Figure 8. Features associated with risk decision making for investigational surgery procedures at individual level

a) MSA patients' median accepted risk regarding surgery willingness

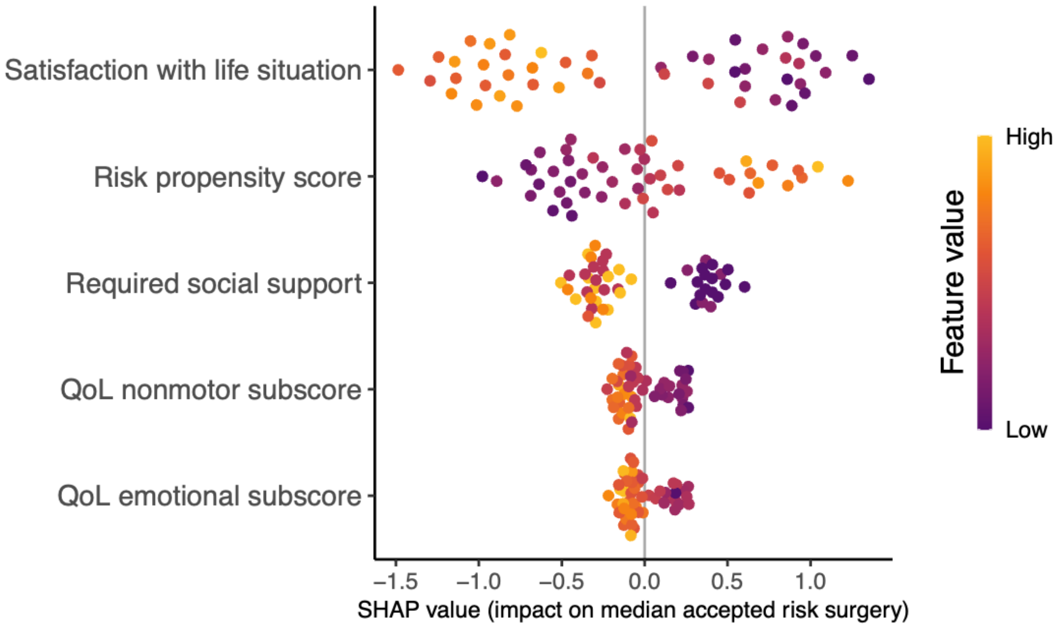

b) MSA patient with the highest therapy-associated risk willingness

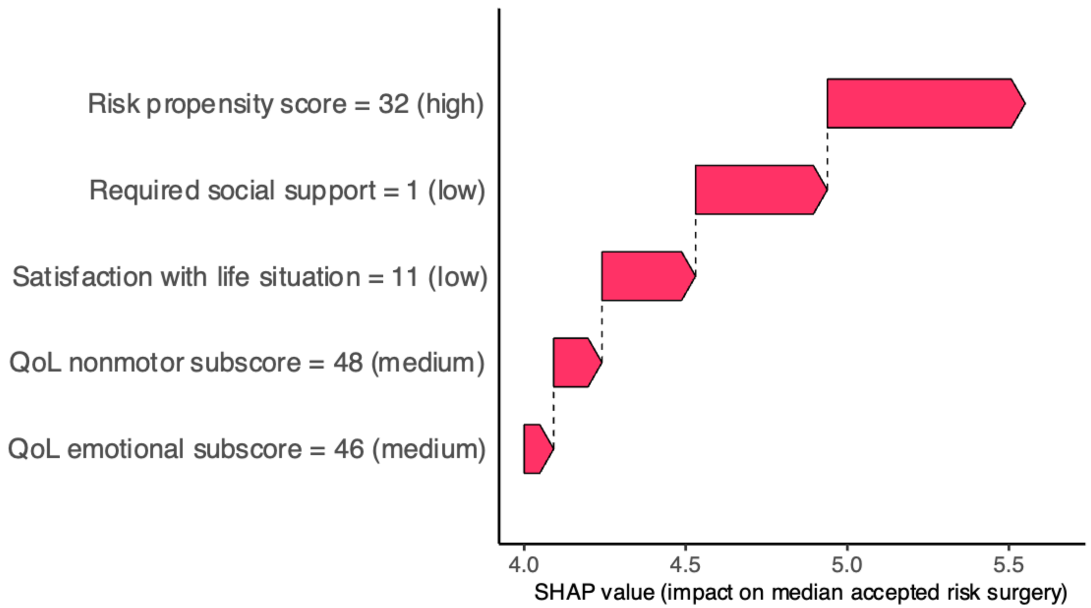

c) MSA patient with medium therapy-associated risk willingness

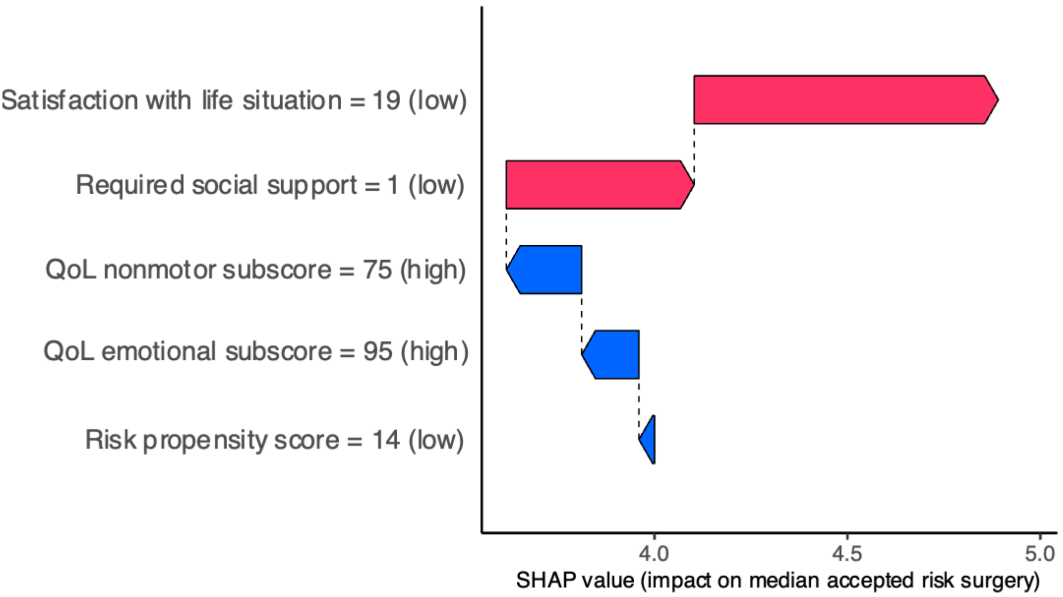

d) MSA patient with the lowest therapy-associated risk willingness

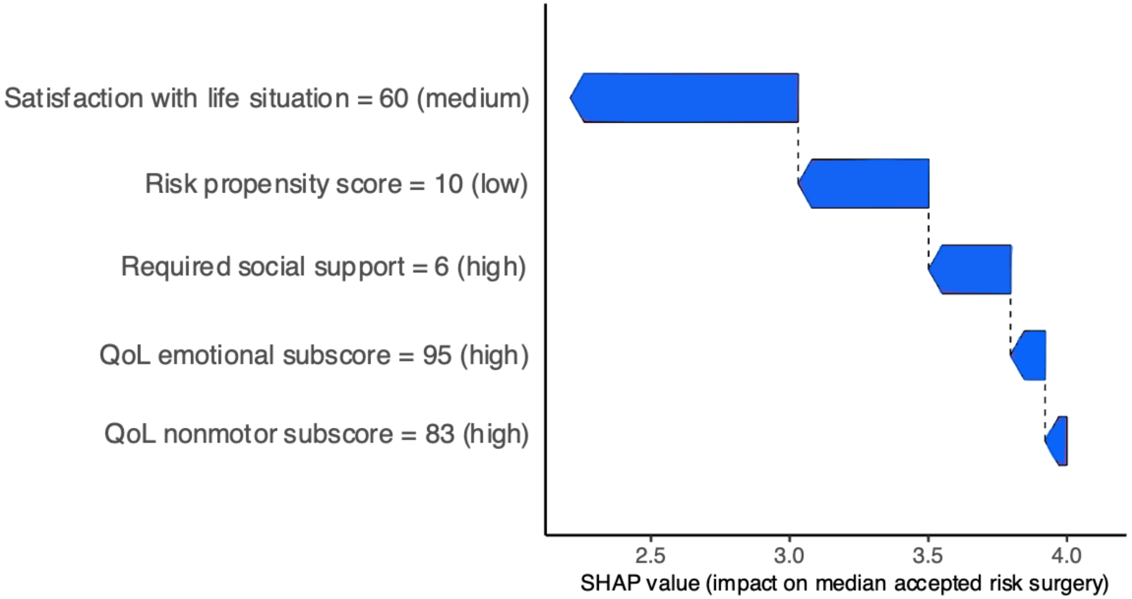

Supplementary Figure 8. Features associated with risk decision making for investigational surgery procedures at individual level

e) PD patients' median accepted risk regarding surgery willingness

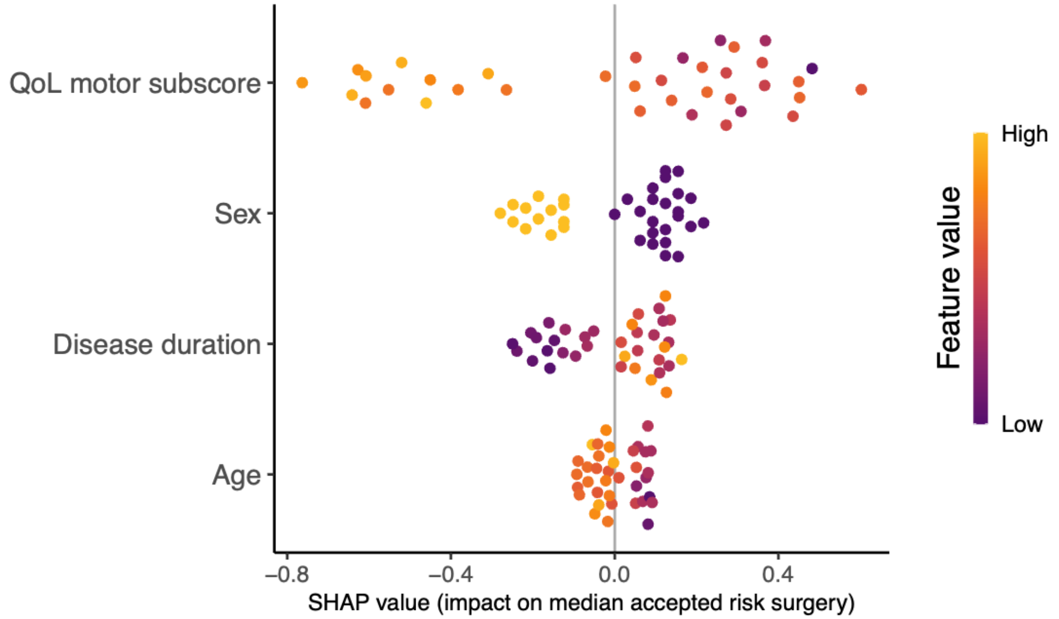

f) PD patient with the highest therapy-associated risk willingness

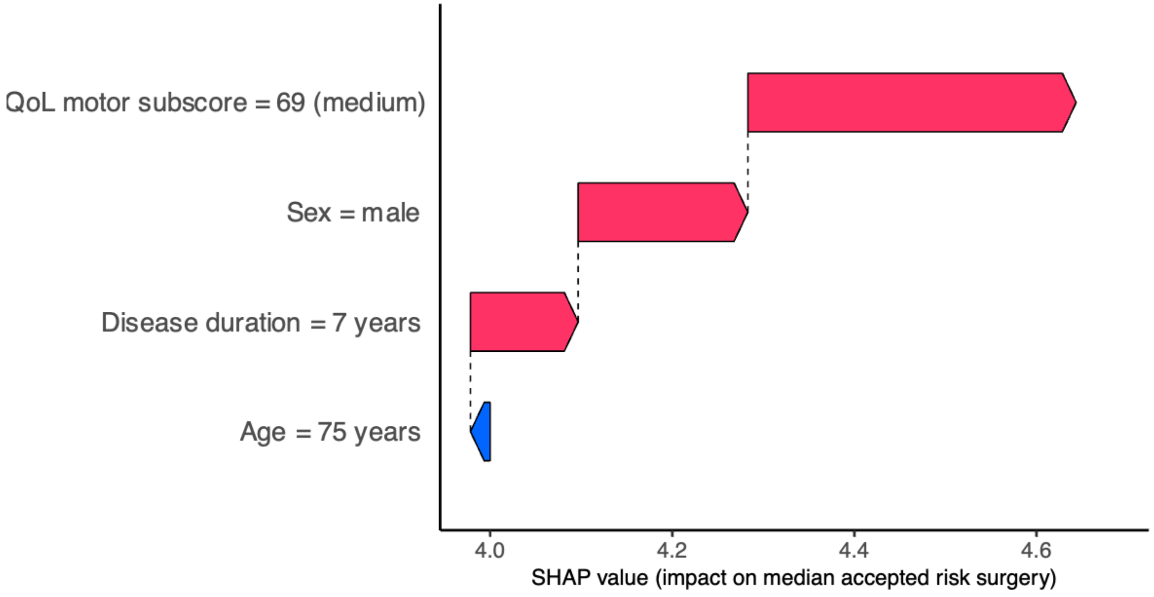

g) PD patient with medium therapy-associated risk willingness

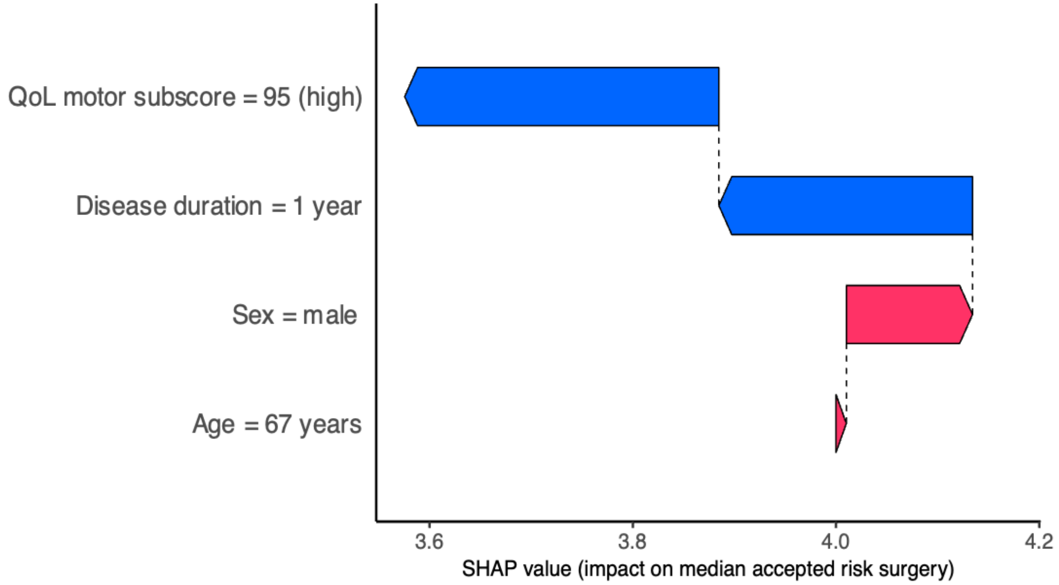

h) PD patient with the lowest therapy-associated risk willingness

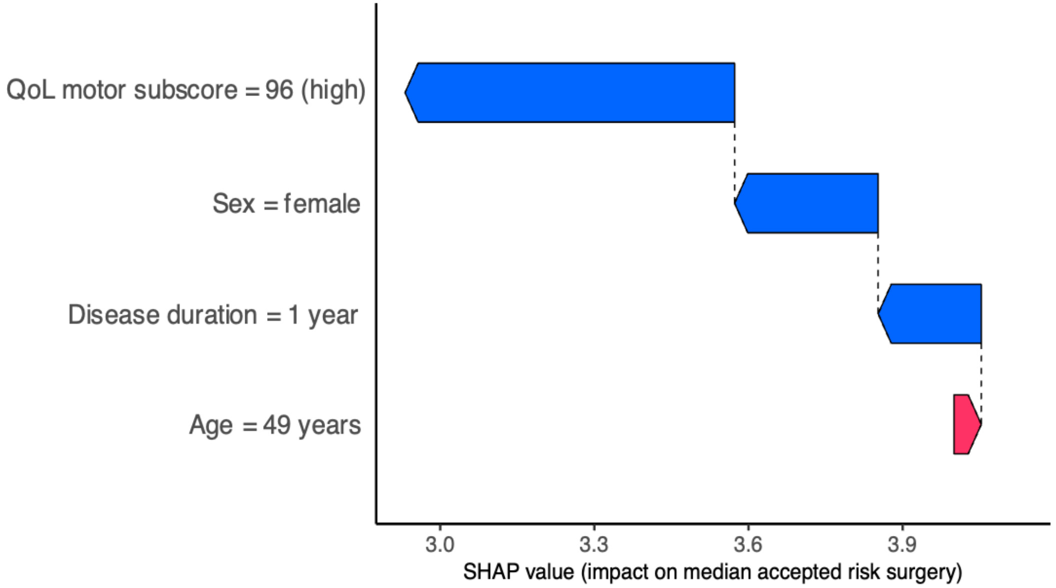

Supplementary Figure 9. Bivariate correlations of clinical and psychosocial features with patients' willingness to take drug related risks

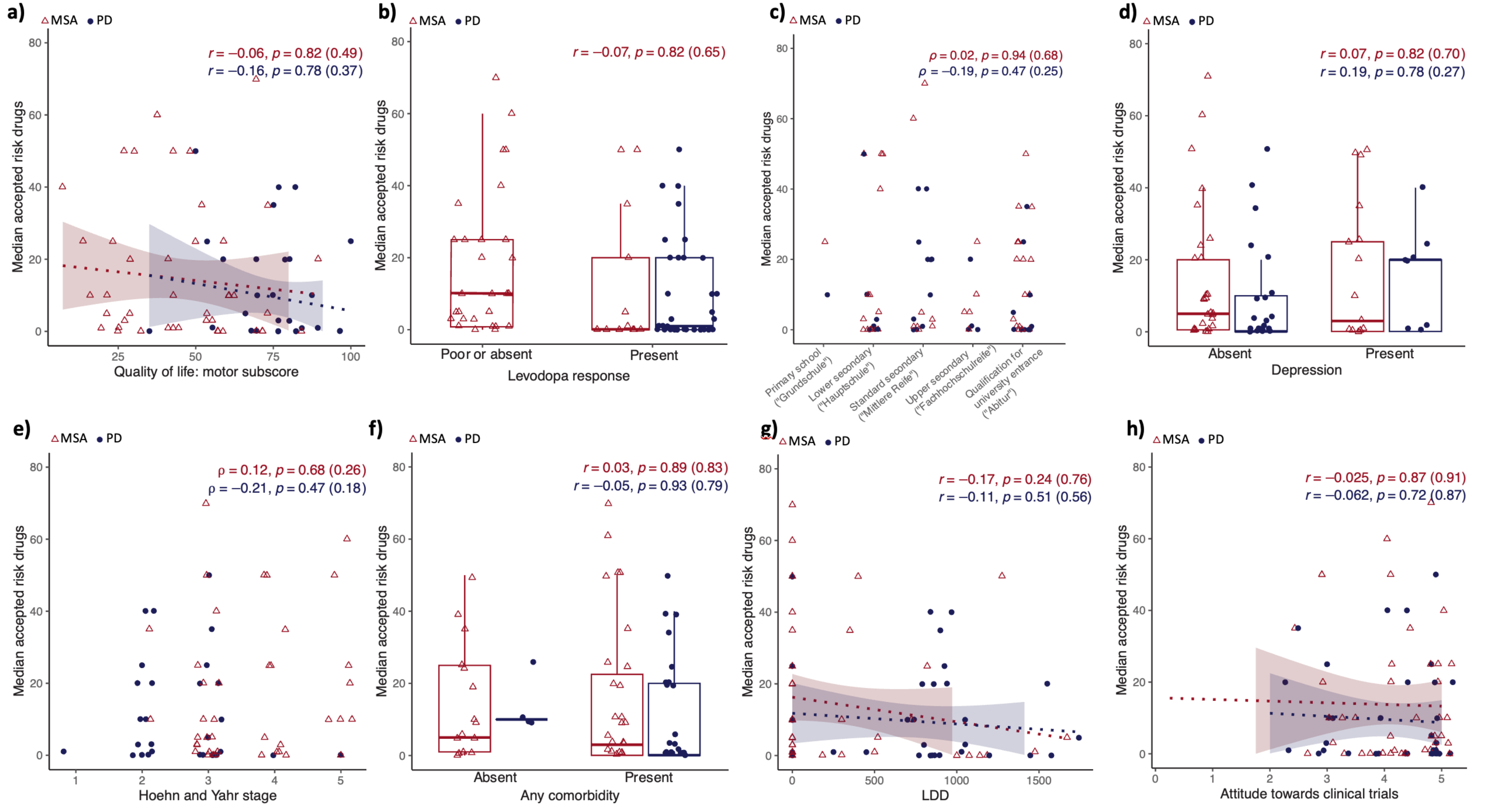

Supplementary Figure 9. Bivariate correlations of clinical and psychosocial features with patients' willingness to take drug related risks

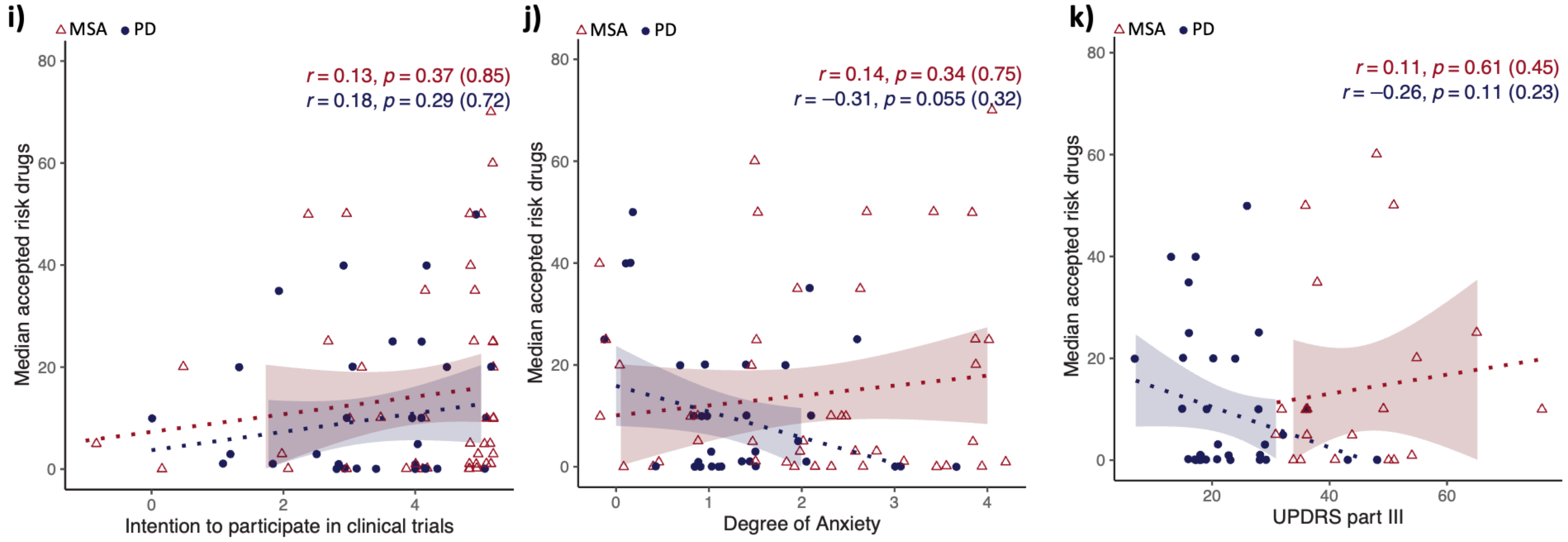

Supplementary Figure 10. Bivariate correlations of clinical and psychosocial features with patients' willingness to take surgery related risks

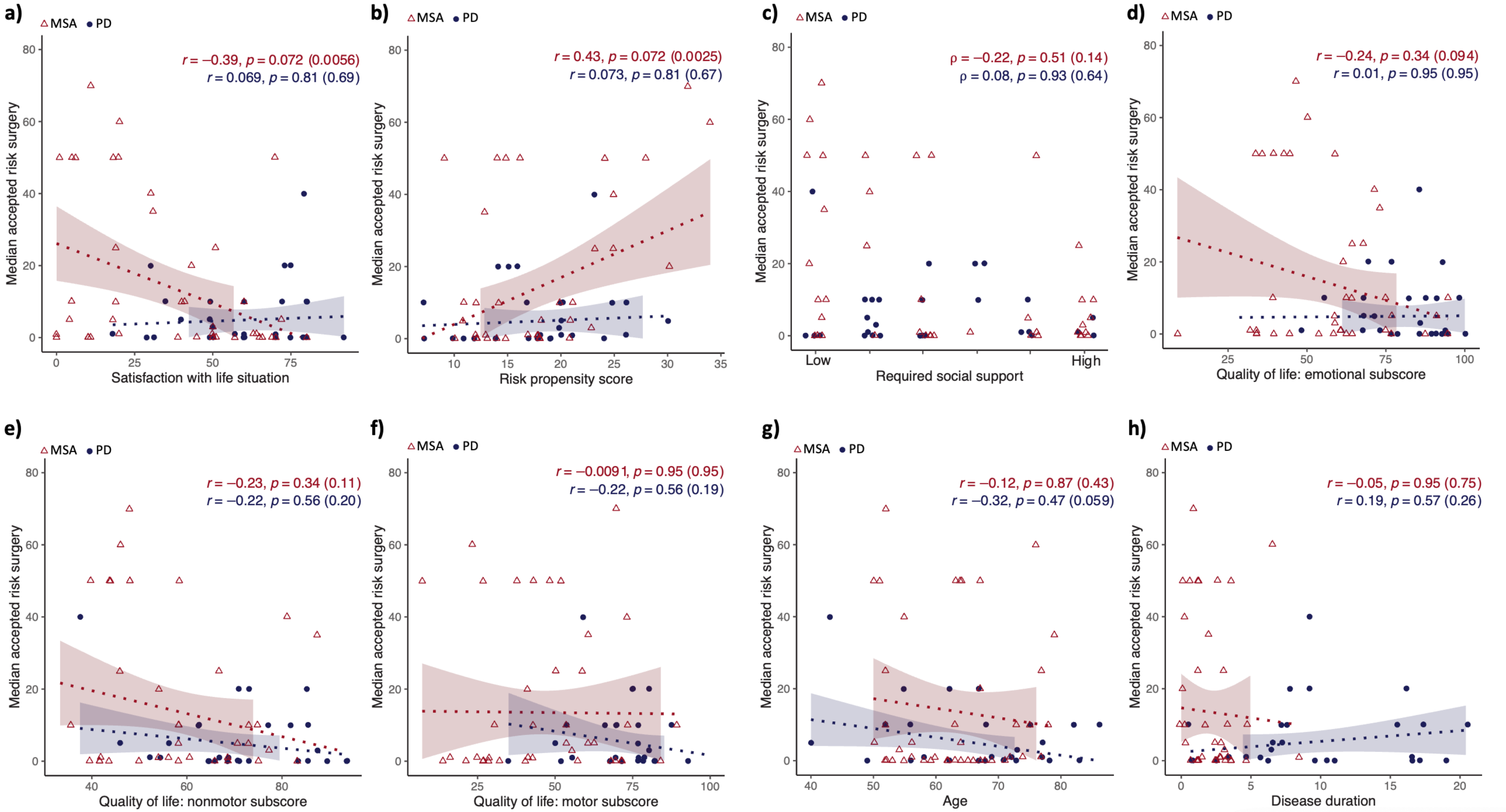

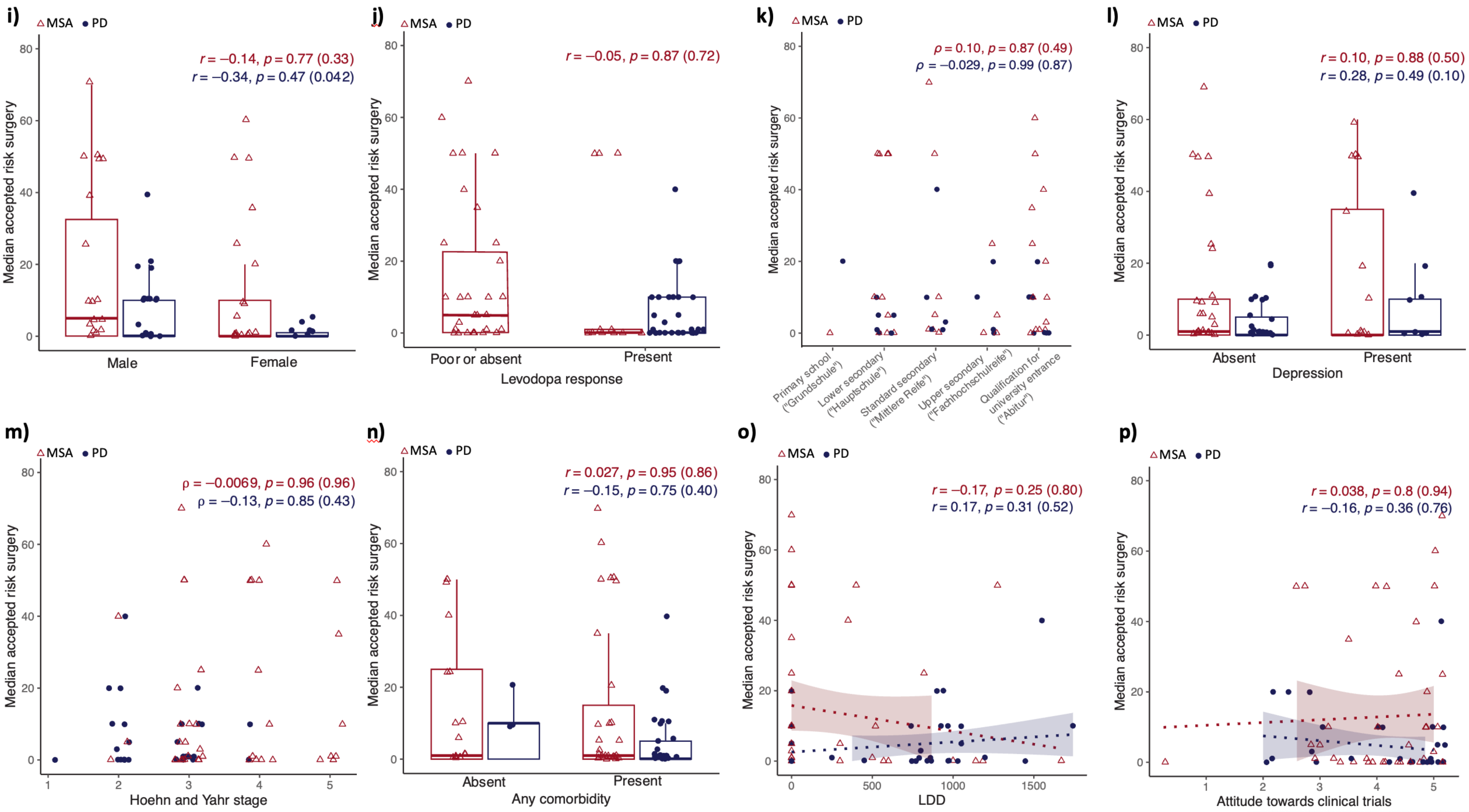
i)
MSA PD
r = –0.14, p = 0.77 (0.33)
r = –0.34, p = 0.47 (0.042)
Median accepted risk surgery
Male
Female
j)
MSA PD
r = –0.05, p = 0.87 (0.72)
Median accepted risk surgery
Poor or absent
Present
Levodopa response
k)
MSA PD
ρ = 0.10, p = 0.87 (0.49)
ρ = –0.029, p = 0.99 (0.87)
Median accepted risk surgery
Primary school ("Grundschule")
Lower secondary ("Hauptschule")
Standard secondary ("Mittlere Reife")
Upper secondary ("Fachhochschulreife")
Qualification for university entrance ("Abitur")
l)
MSA PD
r = 0.10, p = 0.88 (0.50)
r = 0.28, p = 0.49 (0.10)
Median accepted risk surgery
Absent
Present
Depression
m)
MSA PD
ρ = –0.0069, p = 0.96 (0.96)
ρ = –0.13, p = 0.85 (0.43)
Median accepted risk surgery
Hoehn and Yahr stage
n)
MSA PD
r = 0.027, p = 0.95 (0.86)
r = –0.15, p = 0.75 (0.40)
Median accepted risk surgery
Absent
Present
Any comorbidity
o)
MSA PD
r = –0.17, p = 0.25 (0.80)
r = 0.17, p = 0.31 (0.52)
Median accepted risk surgery
LDD
p)
MSA PD
r = 0.038, p = 0.8 (0.94)
r = –0.16, p = 0.36 (0.76)
Median accepted risk surgery
Attitude towards clinical trials

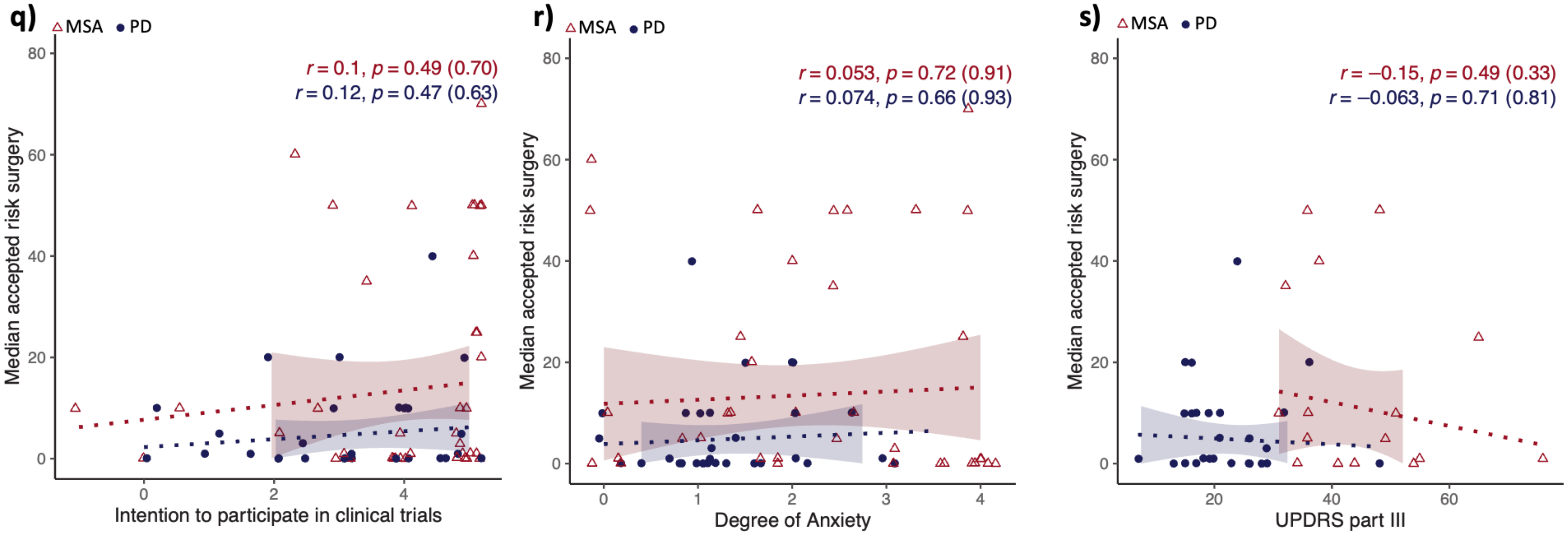
q)
MSA
PD
r = 0.1, p = 0.49 (0.70)
r = 0.12, p = 0.47 (0.63)
Median accepted risk surgery
Intention to participate in clinical trials
r)
MSA
PD
r = 0.053, p = 0.72 (0.91)
r = 0.074, p = 0.66 (0.93)
Median accepted risk surgery
Degree of Anxiety
s)
MSA
PD
r = −0.15, p = 0.49 (0.33)
r = −0.063, p = 0.71 (0.81)
Median accepted risk surgery
UPDRS part III

Supplementary Figure 11. MSA patients' willingness to take risks regarding severity of drug or surgery side effects

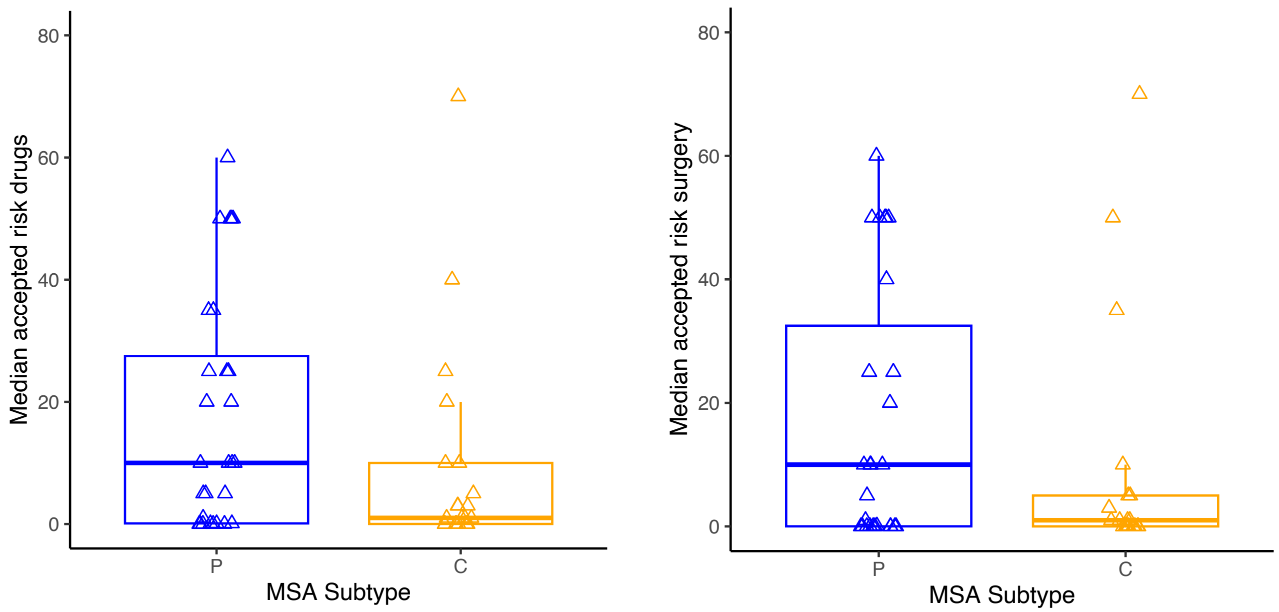

Supplementary File 1.

Attitudes of patients with multiple system atrophy (MSA) towards clinical studies

Questionnaire

Participant-ID

**ID**

Project Management

Professor Wolfgang Oertel
Philipps-University Marburg
Medical Faculty
Department of Neurology
Baldingerstraße
35043 Marburg
Tel: +49 (0)6421 58-63730
oertelw@med.uni-marburg.de

Professor Armin Giese
Ludwig-Maximilians-University Munich
Medical Faculty
Centre for Neuropathology
and Prion Research
Feodor-Lynen-Straße 23
81377 München
Tel.: +49 (0)89 2180-78007
armin.giese@med.uni-muenchen.de

General Instructions

Thank you for agreeing to help us in our research. We would like to know more about your attitude towards participating in a clinical study for people with multiple system atrophy (MSA). Your participation could help to improve future study designs and to implement more clinical trials. Your participation is entirely voluntary and you are free to withdraw at any time without giving any reason.

- All the information that you give us will be **COMPLETELY CONFIDENTIAL** and will not be seen by your doctor. Your doctor is only informed about your participation in the study.
- If you complete and return this questionnaire, we assume that you have consented to us using this information for the purpose of research. If you do not wish to take part then please return the unanswered questionnaire.
- Please answer **ALL** the questions to the best of your ability. You can also ask another person to get assistance with filling in the questionnaire.
- Please fill in the whole questionnaire. For most questions you simply need to tick a box.
- If there are any questions that you would rather not complete then it is fine to leave these blank and go onto the next set of questions.
- Please do not think too long before answering; usually your first inclination is also the best one. There are no right and wrong answers.
- Please do not write your name on the questionnaire. The questionnaire is marked with an ID-number which only we know. This is intended to protect your anonymity.
- Please return the questionnaire to us using the stamped self-addressed envelope.

Thank you very much for helping in our research!

Demographics

sex	❑ female<br>❑ male
Date of birth	19 _______
Which level of educational qualifiaction did you achieve?	❑ no school-leaving qualification<br>❑ GCSE<br>❑ A levels<br>❑ University degree<br>❑ Other: _________________________________

We would like to learn more about your illness and your current therapy now.

When have you been diagnosed with multiple system atrophy (MSA) first?	_________ / _________ (month / year)
Are you currently receiving medical treatment for your MSA?	❑ yes<br>❑ no
Are you currently taking medication in the context of your MSA?	❑ yes<br>Which drug(s)? ___________________________<br>____<br>❑ no, never<br>❑ no, the medication was stopped

Having a health problem can affect a person's quality of life in many ways. In order to understand how your illness affects your life, we are interested which, if any, of the following problems you have experienced. We would also like to know how problematic each has been for you. Please consider your situation in the last four weeks.

<u>Please note that this lists includes many problems that you may never experience.</u>

In the last 4 weeks have you	No Problem	Slight Problem	Moderate Problem	Marked Problem	Extreme Problem	Not appl.
1. Had difficulty moving?	❑	❑	❑	❑	❑	❑
2. Had difficulty walking?	❑	❑	❑	❑	❑	❑
3. Had problems with your balance?	❑	❑	❑	❑	❑	❑

In the last 4 weeks have you	No Problem	Slight Problem	Moderate Problem	Marked Problem	Extreme Problem	Not appl.
4. Had difficulty standing up without support?	❑	❑	❑	❑	❑	❑
5. Had difficulty speaking?	❑	❑	❑	❑	❑	❑
6. Had difficulty with swallowing food?	❑	❑	❑	❑	❑	❑
7. Had to much saliva or drooling?	❑	❑	❑	❑	❑	❑
8. Had difficulty with handwriting?	❑	❑	❑	❑	❑	❑
9. Had difficulty feeding yourself?	❑	❑	❑	❑	❑	❑
10. Had difficulty drinking fluids?	❑	❑	❑	❑	❑	❑
11. Had difficulty dressing yourself?	❑	❑	❑	❑	❑	❑
12. Needed help to go tot he toilet?	❑	❑	❑	❑	❑	❑
13. Had to stop doing things that you liked to do (e.g. your hobbies)?	❑	❑	❑	❑	❑	❑
14. Had difficulty doing things around the house (e.g. housework)?	❑	❑	❑	❑	❑	❑
15. Experienced bladder problems?	❑	❑	❑	❑	❑	❑
16. Experienced problems with constipation?	❑	❑	❑	❑	❑	❑
17. Experienced dizziness when standing up?	❑	❑	❑	❑	❑	❑
18. Suffered from cold hands or feet?	❑	❑	❑	❑	❑	❑

In the last 4 weeks have you	No Problem	Slight Problem	Moderate Problem	Marked Problem	Extreme Problem	Not appl.
19. Experienced pain in your neck or shoulders?	❑	❑	❑	❑	❑	❑
20. Experienced pain elsewhere (e.g. in your legs or your back)?	❑	❑	❑	❑	❑	❑
21. Had difficulty getting comfortable during the night?	❑	❑	❑	❑	❑	❑
22. Had difficulty breathing during the night?	❑	❑	❑	❑	❑	❑
23. Been feeling tired very quickly (without exerting yourself)?	❑	❑	❑	❑	❑	❑
24. Experienced lack of energy?	❑	❑	❑	❑	❑	❑
25. Experienced slowness of thinking?	❑	❑	❑	❑	❑	❑
26. Had difficulty with your concentration (e.g. reading, watching TV)?	❑	❑	❑	❑	❑	❑
27. Felt frustrated?	❑	❑	❑	❑	❑	❑
28. Felt depressed?	❑	❑	❑	❑	❑	❑
29. Experienced a loss of motivation?	❑	❑	❑	❑	❑	❑
30. Been feeling incapable?	❑	❑	❑	❑	❑	❑
31. Worried about the future?	❑	❑	❑	❑	❑	❑
32. Worried about your family?	❑	❑	❑	❑	❑	❑
33. Felt on your own or isolated?	❑	❑	❑	❑	❑	❑

In the last 4 weeks have you	No Problem	Slight Problem	Moderate Problem	Marked Problem	Extreme Problem	Not appl.
34. Exprienced loss of confidence when interacting with others?	❑	❑	❑	❑	❑	❑
35. Felt that your role in family or among friends has changed?	❑	❑	❑	❑	❑	❑
36. Experienced difficulty seeing your friends?	❑	❑	❑	❑	❑	❑
37. Had to give up social activities (e.g. going out for a meal, participating in events)?	❑	❑	❑	❑	❑	❑
38. Had difficulty talking to friends about your illness?	❑	❑	❑	❑	❑	❑
39. Been embarrassed to talk to people?	❑	❑	❑	❑	❑	❑
40. Felt that life has become boring?	❑	❑	❑	❑	❑	❑

Experiencing any illness has an effect on one's life. Please indicate how satisfied you feel overall with your life at the moment by putting a cross on the line between 0 and 100?

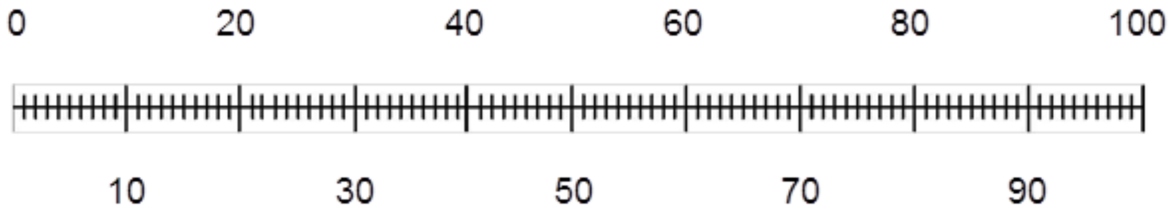

Extremely dissatisfied with my life

Extremely satisfied with my life

We have some questions about your general experience with clinical studies.

Have you already participated in a clinical study?	❑ Yes<br>❑ No
If "**Yes**":<br>Do you remember the study design of this clinical study?	❑ intervention study (e.g. pharmaceuticals, surgery practises)<br>❑ observational study (e.g. questionnaire, clinical examination)<br>❑ Other: _______
If "**No**":<br>Have you already been invited to take part in a clinical study?	❑ Yes, but I could not be included in the study.<br>❑ Yes, but I did not want to take part in the study.<br>❑ No, I have not been invited to take part in a clinical study yet.

For me participating in clinical trials against MSA would be

beneficial	❑	❑	❑	❑	❑	❑	harmful
pleasant	❑	❑	❑	❑	❑	❑	unpleasant
good	❑	❑	❑	❑	❑	❑	bad
desirable	❑	❑	❑	❑	❑	❑	undesirable

Considering your current living conditions, to what extent do you agree with the following statements?

**I itend ...**	**Strongly agree**	**Agree**	**Somewhat Agree**	**Somewhat disagree**	**Disagree**	**Strongly disagree**
… to participate in a clinical study for people with MSA when I am invited to participate.	❑	❑	❑	❑	❑	❑
… to participate in a clinical study for people with MSA when an opportunity arises.	❑	❑	❑	❑	❑	❑

We are interested in the reasons why patients accept to take part in clinical studies. Below are some reasons that may influence your decision to accept to take part in a clinical study. Please answer each question and tick the box that shows most clearly how you feel.

**I would take part in a clinical study only if ...**	**Strongl y agree**	**Agre e**	**Somewha t Agree**	**Somewha t disagree**	**Disagre e**	**Strongly disagre e**
… I thought the study offered the best treatment available.	❑	❑	❑	❑	❑	❑
… I believed the benefits of treatment in the study would out-weigh any side-effects.	❑	❑	❑	❑	❑	❑
… the doctor would recommend me the trial.	❑	❑	❑	❑	❑	❑
… I was given enough information to read about the study.	❑	❑	❑	❑	❑	❑
… I knew that I could leave the study at any time and still be treated.	❑	❑	❑	❑	❑	❑
… I believe that the study will push forward research.	❑	❑	❑	❑	❑	❑
… I feel that others with my illness will benefit from the results of the trial.	❑	❑	❑	❑	❑	❑
… I will be appropriately compensated, e.g. by receiving money.	❑	❑	❑	❑	❑	❑
Further reasons:<br>… ________________________________ _	❑	❑	❑	❑	❑	❑
… ________________________________ _	❑	❑	❑	❑	❑	❑

We are interested in the reasons why patients decline to take part in clinical studies. Below are some reasons that may influence your decision to decline to take part in a clinical study. Please answer each question and tick the box that shows most clearly how you feel.

**I would <u>not</u> take part in a clinical study since**	**Strongly agree**	**Agree**	**Somewhat Agree**	**Somewhat disagree**	**Disagree**	**Strongly disagree**

… it involves a long or expensive journey.	❑	❑	❑	❑	❑	❑
**I would <u>not</u> take part in a clinical study since**	**Strongly agree**	**Agree**	**Somewhat Agree**	**Somewhat disagree**	**Disagree**	**Strongly disagree**
… there is poor public transport.	❑	❑	❑	❑	❑	❑
… my physical constraints make the arrival difficult.	❑	❑	❑	❑	❑	❑
… transport options in my family are lacking.	❑	❑	❑	❑	❑	❑
… others, e.g. family or friends do not support me.	❑	❑	❑	❑	❑	❑
… I believe there are great risks and side-effects.	❑	❑	❑	❑	❑	❑
… I will be subjected to research.	❑	❑	❑	❑	❑	❑
… I do not want to belong to a control group.	❑	❑	❑	❑	❑	❑
… I do not want to endure unpleasant examinations.	❑	❑	❑	❑	❑	❑
… I am not prepared to let doctors carry out procedures on my body such as taking a blood sample and lumbar punctures.	❑	❑	❑	❑	❑	❑
Further reasons: … ___________________	❑	❑	❑	❑	❑	❑
… ___________________	❑	❑	❑	❑	❑	❑

Would you be prepared to participate in a long-term study with either weekly, monthly, semi-anually or annually examinations?

Please tick the appropriate box.

weekly	monthly	Semi-annually	annually	I am not prepared to participate in a study involving examinations.
❑	❑	❑	❑	❑

In general, people often face risks when making financial, career or other life decisions. Overall, how would you place yourself on the following scale from 1-6?

1 ❑	2 ❑	3 ❑	4 ❑	5 ❑	6 ❑
**Extremely Comfortable Taking Risks**					**Extremely Uncomfortable Taking Risks**

Please indicate the extent to which you agree or disagree with the following statement by ticking the option you prefer. Please do not think too long before answering; usually your first inclination is also the best one.

	Strongly agree	Agree	Somewhat Agree	Somewhat disagree	Disagree	Strongly disagree
Safety first.	❑	❑	❑	❑	❑	❑
I do not take risks with my health.	❑	❑	❑	❑	❑	❑
I prefer to avoid risks.	❑	❑	❑	❑	❑	❑
I take risks regularly.	❑	❑	❑	❑	❑	❑
I really dislike not knowing what is going to happen.	❑	❑	❑	❑	❑	❑
I usually view risks as a challenge.	❑	❑	❑	❑	❑	❑

I view myself as a...

1 ❑	2 ❑	3 ❑	4 ❑	5 ❑	6 ❑
... **Risk avoider**					... **Risk seeker**

This last part of the questionnaire deals with side effects and risks which might be associated with your participation in a clinical study. We would like to know more about the risk-willingness of patients with MSA. Our results could help to improve future study designs and to implement more clinical trials. Please do not think too long before answering; usually your first inclination is also the best one. There are no right or wrong answers.

In the first part, we assess the willingness to take drugs despite possible side effects. In the second part we assess the willingness to allow surgeons to perform operations despite possible complications.
In both parts, possible side effects / complications will be presented to you, respectively. Afterwards, we assess your willingness for the scenario.

side effects of drugs

Below, we present you possible physical side effects of drugs. Imagine you take a drug in the course of a clinical study and you suffer from the respective side effects **permanently**.

Please tick **the three most severe side effects** you can think of:

❑ dizziness
❑ sleep disturbances
❑ constipation
❑ diarrhoea
❑ visual disturbances
❑ loss of taste
❑ hair loss
❑ hearing loss
❑ itching
❑ muscle cramps
❑ racing heart
❑ headaches
❑ nausea/vomiting

Below, we present you possible mental side effects of drugs. Imagine you take a drug in the course of a clinical study and you suffer from the respective side effects **permanently**.

Please tick **the three most severe side effects** you can think of:

❑ tiredness
❑ sadness
❑ nervousness
❑ personality changes
❑ aggressiveness
❑ listlessness
❑ hallucinations
❑ confusion
❑ memory loss

Imagine a new (make believe) pill is now available for all your MSA related problems. Your doctor advises you that if you take the pill today and if it works it stops your MSA immediately. However, if you take the pill and it does not work **the most severe side effects you can think of can occur strongly and permanently**. Your doctor has no way of predicting which patients will be cured by this new (make believe) pill, and will support whatever decision you make. We want to know what you think about this pill.

Please circle yes or no for every question.

**Would you take this pill right now if you knew...**	yes	no
... it had a **100%** chance of cure and the most severe side effects you can think of occurred permanently at **0%**	❑	❑
... it had a **99.9999%** chance of cure and the most severe side effects you can think of occurred permanently **1 in 1 million (0.0001%)**	❑	❑
... it had a **99.999%** chance of cure and the most severe side effects you can think of occurred permanently **1 in 100,000 (0.001%)**	❑	❑
... it had a **99.99%** chance of cure and the most severe side effects you can think of occurred permanently **1 in 10,000 (0.01%)**	❑	❑
... it had a **99.9**% chance of cure and the most severe side effects you can think of occurred permanently **1 in 1000 (0.1%)**	❑	❑
... it had a **99%** chance of cure and the most severe side effects you can think of occurred permanently at **1%**	❑	❑
... it had a **97%** chance of cure and the most severe side effects you can think of occurred permanently at **3%**	❑	❑
... it had a **95%** chance of cure and the most severe side effects you can think of occurred permanently at **5%**	❑	❑
... it had a **90%** chance of cure and the most severe side effects you can think of occurred permanently at **10%**	❑	❑
... it had a **80%** chance of cure and the most severe side effects you can think of occurred permanently at **20%**	❑	❑
... it had a **75%** chance of cure and the most severe side effects you can think of occurred permanently at **25%**	❑	❑
... it had a **65%** chance of cure and the most severe side effects you can think of occurred permanently at **35%**	❑	❑
... it had a **60%** chance of cure and the most severe side effects you can think of occurred permanently at **40%**	❑	❑
... it had a **50%** chance of cure and the most severe side effects you can think of occurred permanently at **50%**	❑	❑
... it had a **40%** chance of cure and the most severe side effects you can think of occurred permanently at **60%**	❑	❑
... it had a **30%** chance of cure and the most severe side effects you can think of occurred permanently at **70%**	❑	❑
... it had a **20%** chance of cure and the most severe side effects you can think of occurred permanently at **80%**	❑	❑
... it had a **10%** chance of cure and the most severe side effects you can think of occurred permanently at **90%**	❑	❑

Below, we present you again possible physical side effects of drugs. Imagine you take a drug in the course of a clinical study and you suffer from the respective side effects **permanently**.

However, please tick **the three most bearable side effects** you can think of:

- ❑ dizziness
- ❑ sleep disturbances
- ❑ constipation
- ❑ diarrhoea
- ❑ visual disturbances
- ❑ loss of taste
- ❑ hair loss
- ❑ hearing loss
- ❑ itching
- ❑ muscle cramps
- ❑ racing heart
- ❑ headaches
- ❑ nausea/vomiting

Below, we present you possible mental side effects of drugs. Imagine you take a drug in the course of a clinical study and you suffer from the respective side effects **permanently**.

Please tick **the three most bearable side effects** you can think of:

- ❑ tiredness
- ❑ sadness
- ❑ nervousness
- ❑ personality changes
- ❑ aggressiveness
- ❑ listlessness
- ❑ hallucinations
- ❑ confusion
- ❑ memory loss

Imagine a new (make believe) pill is now available for all your MSA related problems. Your doctor advises you that if you take the pill today and if it works it stops your MSA immediately. However, if you take the pill and it does not work **the most bearable side effects you can think of can occur strongly and permanently**. Your doctor has no way of predicting which patients will be cured by this new (make believe) pill, and will support whatever decision you make. We want to know what you think about this pill.

Please circle yes or no for every question.

**Would you take this pill right now if you knew...**	yes	no
... it had a **100%** chance of cure and the most bearable side effects you can think of occurred permanently at **0%**	❑	❑
... it had a **99.9999%** chance of cure and the most bearable side effects you can think of occurred permanently **1 in 1 million (0.0001%)**	❑	❑
... it had a **99.999%** chance of cure and the most bearable side effects you can think of occurred permanently **1 in 100,000 (0.001%)**	❑	❑
... it had a **99.99%** chance of cure and the most bearable side effects you can think of occurred permanently **1 in 10,000 (0.01%)**	❑	❑
... it had a **99.9**% chance of cure and the most bearable side effects you can think of occurred permanently **1 in 1000 (0.1%)**	❑	❑
... it had a **99%** chance of cure and the most bearable side effects you can think of occurred permanently at **1%**	❑	❑
... it had a **97%** chance of cure and the most bearable side effects you can think of occurred permanently at **3%**	❑	❑
... it had a **95%** chance of cure and the most bearable side effects you can think of occurred permanently at **5%**	❑	❑
... it had a **90%** chance of cure and the most bearable side effects you can think of occurred permanently at **10%**	❑	❑
... it had a **80%** chance of cure and the most bearable side effects you can think of occurred permanently at **20%**	❑	❑
... it had a **75%** chance of cure and the most bearable side effects you can think of occurred permanently at **25%**	❑	❑
... it had a **65%** chance of cure and the most bearable side effects you can think of occurred permanently at **35%**	❑	❑
... it had a **60%** chance of cure and the most bearable side effects you can think of occurred permanently at **40%**	❑	❑
... it had a **50%** chance of cure and the most bearable side effects you can think of occurred permanently at **50%**	❑	❑
... it had a **40%** chance of cure and the most bearable side effects you can think of occurred permanently at **60%**	❑	❑
... it had a **30%** chance of cure and the most bearable side effects you can think of occurred permanently at **70%**	❑	❑
... it had a **20%** chance of cure and the most bearable side effects you can think of occurred permanently at **80%**	❑	❑
... it had a **10%** chance of cure and the most bearable side effects you can think of occurred permanently at **90%**	❑	❑

Imagine a new (make believe) pill is now available for all your MSA related problems. Your doctor advises you that if you take the pill today and if it works it stops your MSA immediately. However, if you take the pill and it does not work it causes death by heart attacks or strokes. Your doctor has no way of predicting which patients will be cured by this new (make believe) pill, and will support whatever decision you make. We want to know what you think about this pill.

Please circle yes or no for every question.

**Would you take this pill right now if you knew...**	yes	no
... it had a **100%** chance of cure and **0%** risk of causing death?	❑	❑
... it had a **99.9999%** chance of cure and a **1 in 1 million (0.0001%)** risk of causing death?	❑	❑
... it had a **99.999%** chance of cure and a **1 in 100,000 (0.001%)** risk of causing death?	❑	❑
... it had a **99.99%** chance of cure and a **1 in 10,000 (0.01%)** risk of causing death?	❑	❑
... it had a **99.9%** chance of cure and a **1 in 1000 (0.1%)** risk of causing death?	❑	❑
... it had a **99%** chance of cure and a **1%** risk of causing death?	❑	❑
... it had a **97%** chance of cure and a **3%** risk of causing death?	❑	❑
... it had a **95%** chance of cure and a **5%** risk of causing death?	❑	❑
... it had a **90%** chance of cure and a **10%** risk of causing death?	❑	❑
... it had a **80%** chance of cure and a **20%** risk of causing death?	❑	❑
... it had a **75%** chance of cure and a **25%** risk of causing death?	❑	❑
... it had a **65%** chance of cure and a **35%** risk of causing death?	❑	❑
... it had a **60%** chance of cure and a **40%** risk of causing death?	❑	❑
... it had a **50%** chance of cure and a **50%** risk of causing death?	❑	❑
... it had a **40%** chance of cure and a **60%** risk of causing death?	❑	❑
... it had a **30%** chance of cure and a **70%** risk of causing death?	❑	❑
... it had a **20%** chance of cure and a **80%** risk of causing death?	❑	❑
... it had a **10%** chance of cure and a **90%** risk of causing death?	❑	❑

Below, we present you possible complications after surgery. Imagine surgery is performed in the course of a clinical study and you suffer from the respective complications **permanently**.

Please tick **the three most severe complications** you can think of:

- ❑ dizziness
- ❑ paralysis in the arms or legs
- ❑ epileptic seizures
- ❑ visual disturbances
- ❑ loss of taste
- ❑ hearing loss
- ❑ headaches
- ❑ nausea/vomiting
- ❑ secondary bleeding
- ❑ memory loss

Imagine you participate in a clinical study, in which you have a new (make believe) brain surgery which is able to stop your MSA. Unfortunately your doctor has no way of predicting which patients will be cured by this new (make believe) operation. After this operation of several hours, there is the risk that **the most severe complications you can think of can occur strongly and permanently**.

Please circle yes or no for every question.

**Would you allow the surgeon to perform the operation if …**	yes	no
... it had a **100%** chance of cure and the most severe complications you can think of occurred permanently at **0%**	❑	❑
... it had a **99.9999%** chance of cure and the most severe complications you can think of occurred permanently **1 in 1 million (0.0001%)**	❑	❑
... it had a **99.999%** chance of cure and the most severe complications you can think of occurred permanently **1 in 100,000 (0.001%)**	❑	❑
... it had a **99.99%** chance of cure and the most severe complications you can think of occurred permanently **1 in 10,000 (0.01%)**	❑	❑
... it had a **99.9**% chance of cure and the most severe complications you can think of occurred permanently **1 in 1000 (0.1%)**	❑	❑
... it had a **99%** chance of cure and the most severe complications you can think of occurred permanently at **1%**	❑	❑
... it had a **97%** chance of cure and the most severe complications you can think of occurred permanently at **3%**	❑	❑
... it had a **95%** chance of cure and the most severe complications you can think of occurred permanently at **5%**	❑	❑
... it had a **90%** chance of cure and the most severe complications you can think of occurred permanently at **10%**	❑	❑
... it had a **80%** chance of cure and the most severe complications you can think of occurred permanently at **20%**	❑	❑
... it had a **75%** chance of cure and the most severe complications you can think of occurred permanently at **25%**	❑	❑
... it had a **65%** chance of cure and the most severe complications you can think of occurred permanently at **35%**	❑	❑
... it had a **60%** chance of cure and the most severe complications you can think of occurred permanently at **40%**	❑	❑
... it had a **50%** chance of cure and the most severe complications you can think of occurred permanently at **50%**	❑	❑
... it had a **40%** chance of cure and the most severe complications you can think of occurred permanently at **60%**	❑	❑
... it had a **30%** chance of cure and the most severe complications you can think of occurred permanently at **70%**	❑	❑
... it had a **20%** chance of cure and the most severe complications you can think of occurred permanently at **80%**	❑	❑
... it had a **10%** chance of cure and the most severe complications you can think of occurred permanently at **90%**	❑	❑

Below, we present you again possible complications after surgery. Imagine surgery is performed in the course of a clinical study and you suffer from the respective complications **permanently**.

However, please tick **the three most bearable complications** you can think of:

❑ dizziness	❑ hearing loss
❑ paralysis in the arms or legs	❑ headaches
❑ epileptic seizures	❑ nausea/vomiting
❑ visual disturbances	❑ secondary bleeding
❑ loss of taste	❑ memory loss

Imagine you participate in a clinical study, in which you have a new (make believe) brain surgery which is able to stop your MSA. Unfortunately your doctor has no way of predicting which patients will be cured by this new (make believe) operation. After this operation of several hours, there is the risk **that the most bearable complications you can think of can occur strongly and permanently**.

Please circle yes or no for every question.

**Would you allow the surgeon to perform the operation if …**	yes	no
... it had a **100%** chance of cure and the most bearable complications you can think of occurred permanently at **0%**	❑	❑
... it had a **99.9999%** chance of cure and the most bearable complications you can think of occurred permanently **1 in 1 million (0.0001%)**	❑	❑
... it had a **99.999%** chance of cure and the most bearable complications you can think of occurred permanently **1 in 100,000 (0.001%)**	❑	❑
... it had a **99.99%** chance of cure and the most bearable complications you can think of occurred permanently **1 in 10,000 (0.01%)**	❑	❑
... it had a **99.9**% chance of cure and the most bearable complications you can think of occurred permanently **1 in 1000 (0.1%)**	❑	❑
... it had a **99%** chance of cure and the most bearable complications you can think of occurred permanently at **1%**	❑	❑
... it had a **97%** chance of cure and the most bearable complications you can think of occurred permanently at **3%**	❑	❑
... it had a **95%** chance of cure and the most bearable complications you can think of occurred permanently at **5%**	❑	❑
... it had a **90%** chance of cure and the most bearable complications you can think of occurred permanently at **10%**	❑	❑
... it had a **80%** chance of cure and the most bearable complications you can think of occurred permanently at **20%**	❑	❑
... it had a **75%** chance of cure and the most bearable complications you can think of occurred permanently at **25%**	❑	❑
... it had a **65%** chance of cure and the most bearable complications you can think of occurred permanently at **35%**	❑	❑
... it had a **60%** chance of cure and the most bearable complications you can think of occurred permanently at **40%**	❑	❑
... it had a **50%** chance of cure and the most bearable complications you can think of occurred permanently at **50%**	❑	❑
... it had a **40%** chance of cure and the most bearable complications you can think of occurred permanently at **60%**	❑	❑
... it had a **30%** chance of cure and the most bearable complications you can think of occurred permanently at **70%**	❑	❑
... it had a **20%** chance of cure and the most bearable complications you can think of occurred permanently at **80%**	❑	❑
... it had a **10%** chance of cure and the most bearable complications you can think of occurred permanently at **90%**	❑	❑

Imagine you have a new (make believe) brain surgery of several hours which is able to stop your MSA. Your doctor advises you that if the operation is performed today and it works it stops your MSA immediately. However, there is also the risk that you might die from this operation. Your doctor has no way of predicting which patients will be cured by this new (make believe) operation, and will support whatever decision you make. We want to know what you think about this operation.

Please circle yes or no for every question.

**Would you allow the surgeon to perform the operation if …**	yes	no
... it had a **100%** chance of cure and **0%** risk of causing death?	❑	❑
... it had a **99.9999%** chance of cure and a **1 in 1 million (0.0001%)** risk of causing death?	❑	❑
... it had a **99.999%** chance of cure and a **1 in 100,000 (0.001%)** risk of causing death?	❑	❑
... it had a **99.99%** chance of cure and a **1 in 10,000 (0.01%)** risk of causing death?	❑	❑
... it had a **99.9%** chance of cure and a **1 in 1000 (0.1%)** risk of causing death?	❑	❑
... it had a **99%** chance of cure and a **1%** risk of causing death?	❑	❑
... it had a **97%** chance of cure and a **3%** risk of causing death?	❑	❑
... it had a **95%** chance of cure and a **5%** risk of causing death?	❑	❑
... it had a **90%** chance of cure and a **10%** risk of causing death?	❑	❑
... it had a **80%** chance of cure and a **20%** risk of causing death?	❑	❑
... it had a **75%** chance of cure and a **25%** risk of causing death?	❑	❑
... it had a **65%** chance of cure and a **35%** risk of causing death?	❑	❑
... it had a **60%** chance of cure and a **40%** risk of causing death?	❑	❑
... it had a **50%** chance of cure and a **50%** risk of causing death?	❑	❑
... it had a **40%** chance of cure and a **60%** risk of causing death?	❑	❑
... it had a **30%** chance of cure and a **70%** risk of causing death?	❑	❑
... it had a **20%** chance of cure and a **80%** risk of causing death?	❑	❑
... it had a **10%** chance of cure and a **90%** risk of causing death?	❑	❑

Did you have assistance in filling out the questionnaire?

❑ no

❑ yes

(e.g. having the questionnaire read to you or ticking the boxes by another person)

In your opinion, what has received too little coverage in the questionnaire or was not assessed at all? You can record your suggestions and thoughts as well as additional information below.

Please fill in today's date (date of filling in the questionnaire)**:** **___. ___. 201__**

Please return the questionnaire to us using the stamped self-addressed envelope.

Thank you very much for helping in our research!

Supplementary File 2.

STROBE Statement—checklist of items that should be included in reports of observational studies

	Item No	Recommendation	Reported on page No.
**Title and abstract**	1	(*a*) Indicate the study's design with a commonly used term in the title or the abstract	3
		(*b*) Provide in the abstract an informative and balanced summary of what was done and what was found	3
**Introduction**			
Background/rationale	2	Explain the scientific background and rationale for the investigation being reported	5
Objectives	3	State specific objectives, including any prespecified hypotheses	5
**Methods**			
Study design	4	Present key elements of study design early in the paper	5
Setting	5	Describe the setting, locations, and relevant dates, including periods of recruitment, exposure, follow-up, and data collection	5, Supplement 1
Participants	6	(*a*) *Cohort study*—Give the eligibility criteria, and the sources and methods of selection of participants. Describe methods of follow-up	NA
		Case-control study—Give the eligibility criteria, and the sources and methods of case ascertainment and control selection. Give the rationale for the choice of cases and controls	NA
		Cross-sectional study—Give the eligibility criteria, and the sources and methods of selection of participants	5
		(*b*) *Cohort study*—For matched studies, give matching criteria and number of exposed and unexposed	NA
		Case-control study—For matched studies, give matching criteria and the number of controls per case	NA
Variables	7	Clearly define all outcomes, exposures, predictors, potential confounders, and effect modifiers. Give diagnostic criteria, if applicable	5-6, Supplement 2-3
Data sources/ measurement	8*	For each variable of interest, give sources of data and details of methods of assessment (measurement). Describe comparability of assessment methods if there is more than one group	5-6, Supplement 2-3
Bias	9	Describe any efforts to address potential sources of bias	6
Study size	10	Explain how the study size was arrived at	5
Quantitative variables	11	Explain how quantitative variables were handled in the analyses. If applicable, describe which groupings were chosen and why	6-7
Statistical methods	12	(*a*) Describe all statistical methods, including those used to control for confounding	6-7, Supplement 4
		(*b*) Describe any methods used to examine subgroups and interactions	6-7, Supplement 4
		(*c*) Explain how missing data were addressed	6-7, Supplement 4
		(*d*) *Cohort study*—If applicable, explain how loss to follow-up was addressed	NA

		Case-control study—If applicable, explain how matching of cases and controls was addressed	NA
		Cross-sectional study—If applicable, describe analytical methods taking account of sampling strategy	NA
		(*e*) Describe any sensitivity analyses	NA
**Results**			
Participants	13*	(a) Report numbers of individuals at each stage of study—eg numbers potentially eligible, examined for eligibility, confirmed eligible, included in the study, completing follow-up, and analysed	7, Table 1, Supplementary Table 1
		(b) Give reasons for non-participation at each stage	NA
		(c) Consider use of a flow diagram	NA
Descriptive data	14*	(a) Give characteristics of study participants (eg demographic, clinical, social) and information on exposures and potential confounders	7, Table 1, Supplementary Table 1
		(b) Indicate number of participants with missing data for each variable of interest	7, Table 1, Supplementary Table 1
		(c) *Cohort study*—Summarise follow-up time (eg, average and total amount)	NA
Outcome data	15*	*Cohort study*—Report numbers of outcome events or summary measures over time	NA
		Case-control study—Report numbers in each exposure category, or summary measures of exposure	NA
		Cross-sectional study—Report numbers of outcome events or summary measures	7, Table 1, Supplementary Table 1
Main results	16	(*a*) Give unadjusted estimates and, if applicable, confounder-adjusted estimates and their precision (eg, 95% confidence interval). Make clear which confounders were adjusted for and why they were included	7-9
		(*b*) Report category boundaries when continuous variables were categorized	NA
		(*c*) If relevant, consider translating estimates of relative risk into absolute risk for a meaningful time period	NA
Other analyses	17	Report other analyses done—eg analyses of subgroups and interactions, and sensitivity analyses	7-9, Supplementary Figures 1-10
**Discussion**			
Key results	18	Summarise key results with reference to study objectives	9
Limitations	19	Discuss limitations of the study, taking into account sources of potential bias or imprecision. Discuss both direction and magnitude of any potential bias	10-11
Interpretation	20	Give a cautious overall interpretation of results considering objectives, limitations, multiplicity of analyses, results from similar studies, and other relevant evidence	9-11
Generalisability	21	Discuss the generalisability (external validity) of the study results	9-11

**Other information**			
Funding	22	Give the source of funding and the role of the funders for the present study and, if applicable, for the original study on which the present article is based	13

*Give information separately for cases and controls in case-control studies and, if applicable, for exposed and unexposed groups in cohort and cross-sectional studies.

Note: An Explanation and Elaboration article discusses each checklist item and gives methodological background and published examples of transparent reporting. The STROBE checklist is best used in conjunction with this article (freely available on the Web sites of PLoS Medicine at http://www.plosmedicine.org/, Annals of Internal Medicine at http://www.annals.org/, and Epidemiology at http://www.epidem.com/). Information on the STROBE Initiative is available at www.strobe-statement.org.
